# General Relationship Between Gibbs Energies and Enthalpies of Solvation, Vaporization, and Complexation for Molecular Compounds

**DOI:** 10.3390/molecules31193562

**Published:** 2026-10-07

**Authors:** Boris N. Solomonov, Mikhail I. Yagofarov

**Affiliations:** Department of Physical Chemistry, Kazan Federal University, Kremlevskaya Str. 18, 420008 Kazan, Russia

**Keywords:** compensation phenomenon, solution thermodynamics, hydrogen bonding, charge transfer, molecular complexation, supramolecular recognition, vaporization

## Abstract

The Gibbs equation describes the relationship between the enthalpy (∆*H*), entropy (∆*S*), and Gibbs energy (∆*G*) changes in physicochemical processes: ∆*G* = ∆*H* − *T*∆*S*. It remains a cornerstone of chemical thermodynamics, yet it has features that need to be understood. The linear correlations between ∆*S* and ∆*H* (or ∆*G* and ∆*H*) are often found across various series of processes. These correlations do not follow from the laws of thermodynamics, thus meaning that there may be additional restrictions on the variation in the thermodynamic potentials. In this study, we attempted to establish the quantitative principles governing the ∆*G* vs. ∆*H* relationship across various processes involving non-covalent interactions at 298.15 K, from solvation and vaporization to molecular complexation and supramolecular recognition. The baseline for the analysis was the linear ∆*G* vs. ∆*H* correlation for solvation in non-associated solute–solvent systems, first noted by Barclay and Butler. The classification of the solute–solvent systems enabled quantification of the deviations associated with conformational flexibility, complexation and solvophobic effects upon solvation. These regularities necessarily meant that the ∆*G* vs. ∆*H* for solution- and gas-phase molecular complexation processes should be described by similar linear relationships, having the same slope as the Barclay–Butler baseline. Furthermore, this framework was applied to *α*-cyclodextrin host–guest systems. The validity of the established relationships was checked against more than 4500 experimental thermodynamic values. They enable the calculation of the enthalpy change from a single equilibrium constant measurement at 298.15 K, with the deviation comparable to an experimental uncertainty. The predictive power does not depend on the process (solvation, vaporization, and complexation), the phase it takes place in (gas, liquid), or the dominating interaction type (collective/pairwise, van der Waals forces/hydrogen bonding/charge transfer), highlighting the uniform character of the discussed relationships.

## 1. Introduction

Equation (1), often called the Gibbs equation, is probably the most commonly used relation in chemical thermodynamics:

∆*G* = ∆*H* − *T*∆*S* (1)

Equation (1) enables the prediction of the course and yield of physicochemical processes, from phase distribution and complexation to chemical reactions. Further, it is an important tool for establishing their driving forces. According to Equation (1), the Gibbs energy change (∆*G*) is expressed through the two independent functions, namely the enthalpy (∆*H*) and entropy (∆*S*). Apart from the manifold direct applications mentioned above, the researchers, who have used Equation (1) for more than a century, also sought the functional dependences between ∆*G*, ∆*H*, and ∆*S*. The ∆*H* vs. ∆*S* relationship has been studied most intensively so far, not only in thermodynamics but also in chemical kinetics.

The studies of the ∆*H* vs. ∆*S* relationship are thought to have started in the 1920s–1930s [1,2,3,4]. We consider Barclay and Butler’s work [3], devoted to desolvation thermodynamics of molecular compounds, as the cornerstone. It has two outcomes, important from our viewpoint. First, in the absence of solvent–solvent and solute–solvent hydrogen bonding (HB), ∆*H* and ∆*S* of desolvation of numerous systems studied at that time lay on the common line. The latter was associated with a Barclay–Butler rule [5,6,7,8]. Second, the data points corresponding to desolvation from ethanol (hydrogen-bonded solvent) significantly deviated from the common relation, particularly from the values for a similar non-hydrogen-bonded solvent, acetone. An exhaustive review of the literature devoted to the ∆*H* vs. ∆*S* relation, also called the compensation relationship, was published in 2001 by Liu and Guo [9]. They mostly focused on the ways to make a distinction between artifactual and physically meaningful ∆*H* vs. ∆*S* correlations and their possible origins. Several attempts to explain the presence of this extrathermodynamic phenomenon, particularly at the molecular level, have been made since then [10,11,12,13].

Over the past 50 years, significant attention has been paid to molecular recognition thermodynamics. The objects of study included crown and aza-crown ethers, cyclodextrins, and proteins. The progress in this field was extensively described in the review by Rekharsky and Inoue [14], who also highlighted the presence of enthalpy–entropy correlations in molecular complex formation thermodynamics. In the last decade, these studies have mostly focused on the protein binding thermodynamics [13,15]. The pharmaceutical’s performance depends on the Gibbs energy of binding to a protein target. At the same time, the efficiency estimates are often made based on the computations of the energy (directly related to the enthalpy) of protein–ligand interactions [16,17]. However, negative entropy offsets the Gibbs energy gain due to the greater binding enthalpy. ∆*G*, ∆*H*, and ∆*S* values are connected through Equation (1), so ∆*G* vs. ∆*H* compensation is of equal interest. Its study might be less demonstrative but more straightforward, as both quantities can be measured directly, contrasting with ∆*S*.

We agree with the assessment of the state-of-the-art level of understanding of the compensation phenomenon: “Enthalpy (∆*H*) − entropy (∆*S*) compensation and activation enthalpy (∆*H^‡^*) − activation entropy (∆*S^‡^*) for equilibrium and kinetic processes, respectively, have been much discussed for nine decades. Many attempts were made to explain the phenomenon. They are empirical, extra-thermodynamic, error-related, involve microscopic thermodynamic processes (statistical reasons), solvent effects, and so forth. Publications on this issue have enriched the literature, but the situation remains complex and unresolved.” [18] Furthermore, we assume that an explanatory approach, together with a common way to analyze the ∆*H* vs. ∆*S* (∆*H* vs. ∆*G*) relations separately for narrow series of processes, currently prevents the generalization and applications of the underlying regularities.

In recent years, we studied the ∆*G* vs. ∆*H* relation for the processes of solvation, vaporization [19,20], and molecular complexation in inert solvents [21] and the gas phase [22], aiming to develop the tools for predicting ∆*H* from ∆*G* and vice versa. In this work, we performed a comparative analysis of the results of works [19,20,21,22] to establish the principles governing the relation between ∆*G*, ∆*H*, and ∆*S* values for the above processes. Based on more than 4500 experimental values, we proved that there are such principles present. Furthermore, we demonstrated that the established regularities can be used for the analysis of intermolecular interactions in more complex processes, such as inclusion compound formation.

## 2. Methodology

### 2.1. Definitions and Framework

Below, we follow the definitions and framework introduced earlier [20,21]. The thermodynamic functions of solvation (Δ_solv_*f*^A/S^) correspond to the transfer of 1 mole of a solute A from the ideal gas phase to the infinitely diluted solution in a solvent S. The thermodynamic functions of vaporization Δ_vap_*f*^A^ correspond to the transfer of 1 mole of A from a neat liquid to the gas phase. They are opposite to the parameters of solvation of A in itself: Δ_vap_*f*^A^ = −Δ_solv_*f*^A/A^. The thermodynamic functions of binding (Δ_bind_*f*^A···B^) correspond to the formation of 1 mole of a complex A···B in reaction described by Equation (2):

(2) A+B⇄A⋯B

We separately consider the complexation process in a solvent S (Δ_bind(S)_*f*^A···B^) and in an ideal gaseous phase (Δ_bind(g)_*f*^A···B^). Throughout the paper, we imply the standard changes in thermodynamic functions and omit the standard state symbol (°) for simplicity. For the dissolved species, the standard state is a hypothetical infinitely diluted solution with a mole fraction of solute x° = 1. For the gaseous species, the standard state is an ideal gas at p° = 1 bar. All the thermodynamic functions refer to *T* = 298.15 K, unless otherwise mentioned.

### 2.2. Classification of Solute–Solvent Systems

One may distinguish several contributions to the thermodynamic functions of solvation, depending on solute and solvent tendencies to hydrogen bonding:

1. No hydrogen bonds are formed upon A solvation in non-hydrogen-bonded S. In this case, nonspecific interactions due to van der Waals forces define the Δ_solv_*f* ^A/S^ value:

Δ_solv_*f*
^A/S^ = Δ_solv(nonsp)_*f*
^A/S^ (3)

2. A^···^S hydrogen bonds form in non-hydrogen-bonded S (e.g., A = triethylamine, S = chloroform; or A = phenol, S = ethyl acetate), so specific interactions between A and S arise:

Δ_solv_*f*
^A/S^ = Δ_solv(nonsp)_*f*
^A/S^ + Δ_solv(sp)_*f*
^A/S^ (4)

3. S^···^S hydrogen bonds are present in a solvent, but A does not form hydrogen bonds (e.g., A = alkane, S = water or methanol). Then the solvophobic effect contributes to Δ_solv_*f* ^A/S^:

Δ_solv_*f*
^A/S^ = Δ_solv(nonsp)_*f*
^A/S^ + Δ_s.e._*f*
^A/S^ (5)

4. A forms hydrogen bonds with an associated solvent S (e.g., A = chloroform and S = methanol). Both solvophobic effect and specific interactions contribute to Δ_solv_*f* ^A/S^:

Δ_solv_*f*
^A/S^ = Δ_solv(nonsp)_*f*
^A/S^ + Δ_solv(sp)_*f*
^A/S^ + Δ_s.e._*f*
^A/S^ (6)

### 2.3. Connection Between the Thermodynamic Parameters of Solvation and Binding

A-S specific interactions in solution are described by Equation (7):

(7) $$A_{\left(S\right)}{+S}_{\left(S\right)}⇄A\cdots S_{\left(S\right)}$$

The thermodynamic functions of specific interactions correspond to the transition of unbound A_(S)_ to an equilibrium mixture A_(S)_ + A···S_(S)_. If the fraction of unbound A in S is *α*^A/S^ and the equilibrium constant for the process (4) is *K*^A···S^, Equations (7)–(9) are valid:

Δ_int(sp)_*G*^A/S^ = *RT* ln *α*^A/S^ (8)

Δ_bind_*G*^A···S^ = −*RT* ln *K*^A···S^ (9)

Δ_int(sp)_*H*^A/S^ = (1 − *α*^A/S^)Δ_bind_*H*^A···S^ (10)

At small *α*^A/S^, Δ_int(sp)_*f*^A/S^ ≈ Δ_bind_*f*^A···S^. One should consider that, while in the present work a mole-fraction scale is adopted, *K*^A···S^ and Δ_bind_*G*^A···S^ are often expressed on a concentration (mol/L) basis. The transition between mole-fraction and concentration-scaled (*c*) binding and solvation Gibbs energies is given by Equations (11) and (12):

(11) $$\Delta_{\mathrm{bind}}G^{A\cdots S}=\Delta_{\mathrm{bind}}G_{c}^{A\cdots S}+RT\ln\left(\frac{V_{m}^{S}}{L\cdot {\mathrm{mol}}^{-1}}\right)$$

(12) $$\Delta_{\mathrm{solv}}G^{A/S}=\Delta_{\mathrm{solv}}G_{c}^{A/S}-RT\ln\left(\frac{V_{m}^{S}}{L\cdot {\mathrm{mol}}^{-1}}\right)$$

### 2.4. Uncertainty Analysis

In Section 3, we demonstrate how the above framework helps to derive the relationships between ∆*G* and ∆*H* of solvation/vaporization and binding in a common form. These relationships enable calculating ∆*H* from a single experimental ∆*G* value at 298.15 K and vice versa. The first option is quite helpful, as the enthalpies are often determined from the temperature dependence of an equilibrium constant, i.e., ∆*G* measurements at several temperatures. Thus, the performance of the established relationships is judged based on the agreement between the calculated (calc.) and the literature (lit.) enthalpy values, expressed as root-mean-square deviation (*RMSD*, Equation (13)), average deviation (*AD*, Equation (14)), relative root-mean-square deviation (*rRMSD*, Equation (15)), and relative average deviation (*rAD*, Equation (16)):

(13) $$RMSD=\sqrt{\frac{\sum {\left(\Delta H\left(\mathrm{calc}.\right)-\Delta H\left(\mathrm{lit}.\right)\right)}^{2}}{N-1}}$$

(14) $$AD=\frac{\sum \left(\Delta H\left(\mathrm{calc}.\right)-\Delta H\left(\mathrm{lit}.\right)\right)}{N}$$

(15) $$rRMSD=\frac{N\cdot \sqrt{\frac{\sum {\left(\Delta H\left(\mathrm{calc}.\right)-\Delta H\left(\mathrm{lit}.\right)\right)}^{2}}{\left(N-1\right)}}}{\sum \Delta H\left(\mathrm{lit}.\right)}$$

(16) $$rAD=\frac{\sum \left(\Delta H\left(\mathrm{calc}.\right)-\Delta H\left(\mathrm{lit}.\right)\right)}{\sum \Delta H\left(\mathrm{lit}.\right)}$$

where *N* is the number of data points taken for comparison. We also use RMSD to characterize the quality of correlations, where it applies to the function (typically ∆*G*).

The above quantities need to be analyzed together with the typical uncertainties of the literature enthalpies and Gibbs energies of solvation/vaporization and molecular complexation.

The Δ_vap_*G* of volatile compounds at 298.15 K, determined from the vapor pressure (*p*_sat_) measurements [23], is typically reproduced within ±0.3 kJ·mol^−1^. A decrease in volatility necessitates measurements at higher temperatures, so extrapolation errors, adjustments for gas imperfection, and thermal decomposition may increase the deviations up to 2–3 kJ·mol^−1^ [24] and more. Δ_solv_*G* of volatile compounds is typically obtained by headspace analysis over diluted solutions. The interlaboratory reproducibility of such experiments at 298.15 K is also typically from ±0.3 to ±0.6 kJ·mol^−1^ [25]. For lower-volatile compounds, extrapolation of vapor–liquid equilibrium data from higher temperatures, coupled with solubility and liquid–liquid distribution data, is employed. Significant errors may accumulate in this case as well.

Most vaporization enthalpies are determined from *p*_sat_ − *T* measurements. *p*_sat_ and *T* error propagation within a typical range of 50 K would lead to an uncertainty of 1–2 kJ·mol^−1^, agreeing with the interlaboratory reproducibility of modern experiments for moderately volatile compounds [24]. For example, the standard deviation of 350 literature Δ_vap_*H* values for 100 aromatic and heteroaromatic compounds was 1.8 kJ·mol^−1^ [26]. Better reproducibility can be attained for calorimetric measurements of Δ_vap_*H* of volatile compounds [27]. On the other hand, adjustment errors, thermal decomposition, and purity issues may cause greater discrepancies. Solvation enthalpies are usually determined as the difference between the solution and vaporization enthalpies. Solution calorimetry contributes negligibly to Δ_solv_*H* error. Greater errors are observed when Δ_solv_*G* is directly measured at several temperatures.

*K*_bind_ in an inert solvent is usually determined by IR, NMR (hydrogen bonding), or UV spectroscopy (charge-transfer (CT) complexation). Abraham [28] evaluated its uncertainty at the level of ±(5–10)%, so typical Δ_bind_*G* uncertainty should also be ±(0.3–0.6) kJ·mol^−1^. Δ_bind_*H* is often obtained from *K*_bind_ − *T* dependence over a ~20 K range, in which error propagation would lead to 2 kJ·mol^−1^ uncertainty. However, the temperature dependence of absorption coefficients, proton donor association, and band overlap can deteriorate the results. For example, for the phenol–pyridine system, 15 literature values from 23.4 to 37.7 kJ·mol^−1^ are available [29]; the average value is 29.1 ± 4.4 kJ·mol^−1^; 8 of them are measured in a tetrachloromethane medium (28.6 ± 4.1 kJ·mol^−1^).

Δ_bind(g)_*f*^A···B^ measurements are more demanding than Δ_bind(S)_*f*^A···B^. Apart from spectroscopy, they may employ *pVT* and heat conductivity determination [30]. Based on the analysis of the reproduced literature data in Ref. [22], we estimated the typical uncertainties of Δ_bind(g)_*G*^A···B^ as ~1 kJ·mol^−1^; for the enthalpy, the standard deviation of the measurements reproduced multiple times (water, alcohols, carboxylic acid dimers, and mixed adducts) varied from 2.5 kJ·mol^−1^ to 5.7 kJ·mol^−1^.

Experimental determination of *K*_bind_ and Δ_bind_*H* to cyclodextrins seems to meet more difficulties compared with HB and CT complexation in an inert solvent. Apart from the techniques mentioned above, one should also mention titration calorimetry as a primary measurement tool [14]. The experiments are typically performed in water, where guest and host ionization, as well as pH- and ionic strength-dependent hydration shell reorganization, may contribute to the measured quantities. Rekharski and Inoue [14] collected numerous data on the thermodynamic functions of binding of cyclodextrins, particularly for α-cyclodextrin. Out of 539 systems reviewed, the uncertainties are provided for 318 Gibbs energies and 393 enthalpies, equaling 0.5 and 1.2 kJ·mol^−1^ on average. However, the authors state that “…it is quite difficult to choose the “best” thermodynamic values or to pick one set of thermodynamic data as “best” when more than two papers deal with the same host–guest combinations under comparable conditions. In such an instance, the only criterion for selecting specific thermodynamic quantities… is an agreement of the results for the same complexation reaction in two or more papers.” Thus, we attempted to evaluate the expected uncertainties of experimental Δ_bind_*G* and Δ_bind_*H* values based on interlaboratory reproducibility of the values obtained under similar conditions (pH difference no more than 1). In the review [14], one can find more than 30 ligands studied multiple times, particularly 1-propanol, 1,3-propanediol, *L*-phenylalanine, phenol, perchlorate anion, 1-pentanol, 1,5-pentanediol, octanoate anion, octanedionate anion, 4-nitrophenol, 2-methyl-2-propanol, methyl orange (acid form), 4-iodophenol, imidazole, hexylammonium cation, 1-hexanol, hexanoate anion, 1,6-hexanediol, heptanoate anion, heptanedionate anion, *D*-glucose, ethanol, cyclohexanol, 4-cyanophenol, 4-chlorophenol, 1-butanol, butanoate anion, 1,4-butanediol, 1-adamantanecarboxylate anion, 1-adamantylammonium cation, and acetic acid. For these ligands, the average standard deviation of Δ_bind_*G* determined in different studies equaled 1.9 kJ·mol^−1^, while for Δ_bind_*H* it was 5.2 kJ·mol^−1^. These estimates agree well with the particular pictures seen for 1-hexanol and 4-nitrophenol, for which 10 and 12 independent measurements are available, respectively. Even under identical conditions, the scatter in the enthalpies sometimes reaches more than 20 kJ·mol^−1^ [14].

## 3. Results and Discussion

In the next subsections, we analyzed the relationships between (1) Δ_solv_*G* and Δ_solv_*H* in molecular solute–solvent systems; (2) Δ_bind_*G* and Δ_bind_*H* in the processes of hydrogen bonding and charge-transfer complexation of organic non-electrolytes in inert solvents and gaseous phases; and (3) Δ_bind_*G* and B_inh_ of organic compounds and polyatomic ions to α-cyclodextrines.

There is a long-standing idea that the form of enthalpy–entropy (or Gibbs energy-enthalpy) relationships depends on the type of interaction mechanism, leading to the variety of Δ*S* vs. Δ*H* (or Δ*G* vs. Δ*H*) correlations observed in different phenomena [4,10,31]. Below we show that, even in distinct physical processes involving diverse intermolecular forces and collective or pairwise interactions, the common regularities in the relationships between Δ*G*, Δ*H*, and Δ*S* hold. These regularities offer a new perspective on the applications of a famous Gibbs equation (∆*G* = ∆*H* − *T*∆*S*).

### 3.1. Features of the Gibbs Equation Applied to Solvation Thermodynamics

As noted above, Barclay and Butler [3] demonstrated that the Δ_solv_*S* and Δ_solv_*H* values in non-associated solute–solvent systems follow a common linear correlation, independent of the molecular structures of the solution components. They highlighted the deviations from the correlation in hydrogen-bonded systems and attributed them to the abnormal surface entropies of cavity formation. Later, Frank and Evans suggested that the deviations seen in aqueous solutions are the manifestation of the hydrophobic effect [5,32]. Fifty years later, it was highlighted again by Lee [33]. Below we demonstrate how the classification described in Section 2.2 enabled us to establish the quantitative relationships between Δ_solv_*G* and Δ_solv_*H* of non-electrolytes in molecular solvents of different nature.

#### 3.1.1. Solutions Without Solute–Solvent and Solvent–Solvent Hydrogen Bonding

In Ref. [19] we established that, for a wide range of aromatic/heteroaromatic, short-chained aliphatic compounds, inert, di- and triatomic gases dissolved in molecular solvents, the Δ_solv_*G* vs. Δ_solv_*H* relationship is described by Equation (17):

(17) $$\Delta_{\mathrm{solv}}G^{A/S}/\left({\mathrm{kJ}\cdot \mathrm{mol}}^{-1}\right)=17.0+0.660\cdot \Delta_{\mathrm{solv}}H^{A/S}/\left({\mathrm{kJ}\cdot \mathrm{mol}}^{-1}\right)$$

*N* = 586, *RMSD* (the root-mean-square deviation) = 1.0 kJ·mol^−1^. However, for 62 long-chained aliphatic hydrocarbons and their derivatives, Equation (17) held at Δ_solv_*H* ≤ −60 kJ·mol^−1^ (*RMSD* = 0.6 kJ·mol^−1^):

(18) $$\Delta_{\mathrm{solv}}G^{A/S}/\left({\mathrm{kJ}\cdot \mathrm{mol}}^{-1}\right)=11.7+0.551\cdot \Delta_{\mathrm{solv}}H^{A/S}/\left({\mathrm{kJ}\cdot \mathrm{mol}}^{-1}\right)$$

Alkyl aromatic compounds were well described by a weighted sum of Equations (17) and (18) [19]. Equations (19) and (20) enable the calculation of Δ_solv_*H* (Δ_vap_*H*) from Δ_solv_*G* (Δ_vap_*G*) at 298.15 K, without calorimetric measurements or determining the temperature dependence of the vapor pressure:

(19) $$\Delta_{\mathrm{vap}}H^{A/S}=\frac{\Delta_{\mathrm{vap}}G^{A/S}+17.0{\;\mathrm{kJ}\cdot \mathrm{mol}}^{-1}}{0.660}$$

(20) $$\Delta_{\mathrm{vap}}H^{A/S}=\frac{\Delta_{\mathrm{vap}}G^{A/S}+11.7{\;\mathrm{kJ}\cdot \mathrm{mol}}^{-1}}{0.551}$$

**Figure 1:**
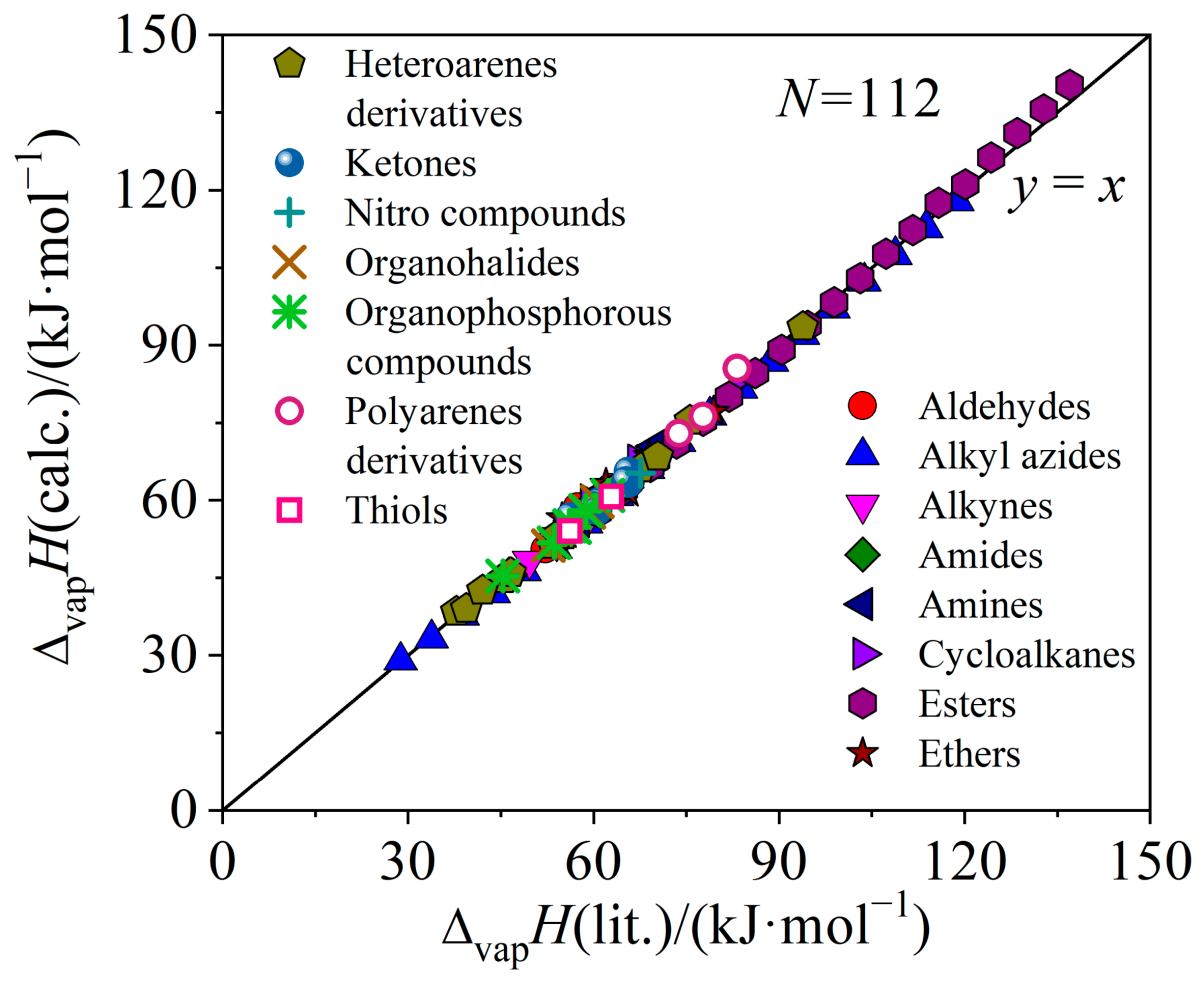
Comparison between the literature vaporization enthalpies of non-hydrogen-bonded organic non-electrolytes and the values calculated from the vaporization Gibbs energies at 298.15 K according to Equations (19) and (20).

We checked the validity of Equations (19) and (20) based on the studies of vaporization of organic compounds [34,35,36,37,38,39,40,41,42,43,44,45,46,47,48] published after our work [19]. The comparison of the literature and calculated Δ_vap_*H* values is provided in Figure 1. The 112 compounds considered include both aromatic and aliphatic organohalides, cycloalkanes, ketones, nitro compounds, aldehydes, ethers, thiols, alkynes, amines, esters, alkyl azides, amides, organophosphorus compounds, and various heteroarenes and polyarene derivatives. The numerical data, together with the references, are listed in Table S1. 

*AD* and *rAD* were −1.1 kJ·mol^−1^ and −1.6%; *RMSD* and *rRMSD* were 1.7 kJ·mol^−1^ and 2.5%. *RMSD* was within the combined uncertainty of Equations (19) and (20), and the experimental determination errors of ΔvapG and ΔvapH (~0.3 kJ·mol^−1^ and ~1.8 kJ·mol^−1^, respectively; see Section 2.4). The uncertainties of the calculated and literature values are within the sizes of the points on the graph.

Nowadays, multiple approaches to the estimation of Δ_solv_*H* (Δ_vap_*H*) are available [49,50,51,52,53]. Equations (17) and (18) also enable the estimation of the vapor pressures of low-volatile compounds at 298.15 K from the estimated Δ_vap_*H* value. Furthermore, one may estimate the vapor pressure temperature dependence from a single *p* measurement or calculated Δ_vap_*H*, combining Equations (17) and (18) with the Clausius–Clapeyron equation [19].

##### Origin of the Deviations Between Aliphatic and Aromatic Compounds

It is reasonable to assume that the deviation between Equations (17) and (18) has an entropic origin, associated with a change in conformational freedom between a gas and a liquid. Indeed, Equations (17) and (18) represent conformationally rigid and flexible molecules. Could this difference stem from the features of intermolecular interactions and their enthalpy? Intermolecular interaction strength in alicyclic hydrocarbons is closer to alkanes than to aromatics, as can be seen when cavity-formation enthalpies or the Hildebrand parameter are compared. However, saturated polycyclic compounds with Δ_vap_*H* > 60 kJ·mol^−1^ (i.e., Δ_solv_*H* in itself < −60 kJ·mol^−1^) obey Equation (17) rather than Equation (18). In Table 1, the literature values of Δ_vap_*G* and Δ_vap_*H* for perhydrochrysene, perhydrophenanthrene, and perhydroacenaphthene are provided, together with Δ_vap_*H* (i.e., −Δ_solv_*H* in itself) calculated from Δ_vap_*G* according to Equations (19) and (20).

Much better agreement of the experimental data on alicyclic compounds with Equation (19) supports the assumption regarding the crucial role of conformational mobility in the difference between Equations (19) and (20).

A semi-quantitative estimate of the internal rotation contribution to the solvation entropy and the difference between Equations (17) and (18) can be made. We compare the solvation Gibbs energies and entropies of rigid and conformationally flexible molecules with equal Δ_solv_*H* values. Δ_solv_*G* (or −Δ_vap_*G*) of a conformationally flexible molecule at 298.15 K is greater (less negative) than for a rigid one, while Δ_solv_*S* (or −Δ_vap_*S*) is lower (more negative). The solvation entropy decrease per each CH_2_-group, compared to rigid molecules, is (0.660 − 0.551) · 5 kJ·mol^−1/^298.15 K ≈ 1.8 J·K^−1^·mol^−1^ (5 kJ·mol^−1^ corresponds to an approximate CH_2_-group increment into the vaporization/solvation enthalpy [56,57]). This deviation may be compared with a difference between the internal rotation contributions to the entropy (*S*_rot_) in liquid and gaseous phases. The rotational barrier height, defining the *S*_rot_ value, is known to be greater in liquids and solutions than in the gas phase, as shown for amides and anilines [58,59,60]. The greater rotational barrier leads to a lower *S*_rot_ value in the case of hindered rotation, agreeing with the lower solvation entropies for flexible molecules. Unfortunately, experimental evaluation of the complete potential energy surface of internal rotation in liquid-phase aliphatic compounds is a non-trivial problem.

Still, one may consider *S*_rot_ corresponding to the rotation along the C-C bond in gaseous and liquid alkanes based on the potential energy vs. dihedral energy dependence. Various torsional potential energy functions (*V*(*φ*) = 0.5Σ*V*_i_(1 − cos(i*φ*))) can be found in the literature for the gaseous alkanes [61], yet their analysis results in quite similar differences between the *S*_rot_ values in the liquid and ideal gas phases. We assumed that the effective parameters *V*_n_ increase by 25% upon transition from the gas to the liquid phase (i.e., Δ*V*_3_ ≈ 3 kJ·mol^−1^), falling within the range seen in Refs. [58,59,60] and considered the *V*(*φ*) functions for the rotation along the central C-C bond in *n*-butane obtained by IR spectroscopy [62], as well as the DFT-derived *V*(*φ*) function averaged for C_2_H_5_-C_2_H_5_, C_2_H_5_-C_3_H_7_, C_2_H_5_-C_4_H_9_, C_3_H_7_-C_3_H_7_, C_2_H_5_-C_5_H_11_, and C_3_H_7_-C_4_H_9_ rotations [61]. Further, we calculated the *S*_rot_ values using the Fourier grid Hamiltonian method [63,64] with varying values of the reduced moment of inertia, starting from 11.0 a.m.u.·Å^2^, corresponding to the C_2_H_5_-C_2_H_5_ rotation [65], up to 1000 a.m.u.·Å^2^, which would correspond to higher alkanes (exact *I*_red_ values would depend on the chain flexibility treatment). The calculation results are presented in Table 2; the calculation details are provided in the Supplementary Materials.

The Δ*S*_rot_ value due to the transition from the gas to the liquid phase is then 2.0 J·K^−1^·mol^−1^. The above model strongly simplifies the rotational barrier form in the liquid phase, as well as ignores the rotation coupling in longer chains. As for the latter, the uncoupled hindered rotor model is known to work well for the thermodynamics of *n*-alkanes, partly due to error cancellation [66]. Nonetheless, the result agrees quite well with the deviation between Equations (17) and (18), i.e., a 1.8 J·K^−1^·mol^−1^ decrease in Δ_solv_*S* per each CH_2_ group.

Equation (17) essentially resembles the Barclay–Butler relation in different coordinates. We will further show that this equation is a clue to the interpretation of the Δ*G* vs. Δ*H* relationship. We shall call the Δ*G* vs. Δ*H* dependence given by Equation (17) the Barclay–Butler line. It should be highlighted that a multitude of solute and solvent molecules of diverse chemical structures were considered with a wide range of the thermodynamic functions of solvation/vaporization (Δ_solv_*H*, Δ_vap_*H*, Δ_solv_*G*, Δ_vap_*G*). The solute–solvent systems exhibit different types of intermolecular interactions (dispersion, dipole-induced, and dipolar interactions), combined in various ways. Still, there is a common Δ*G* vs. Δ*H* relationship.

#### 3.1.2. Solutes Hydrogen-Bonded to Non-Associated Solvents

Non-hydrogen-bonded systems are known to follow fairly simple trends in thermodynamics. However, the introduction of a hydrogen bond makes them far more complicated. Is this the case for solutes hydrogen-bonded to non-associated solvents?

**Figure 2:**
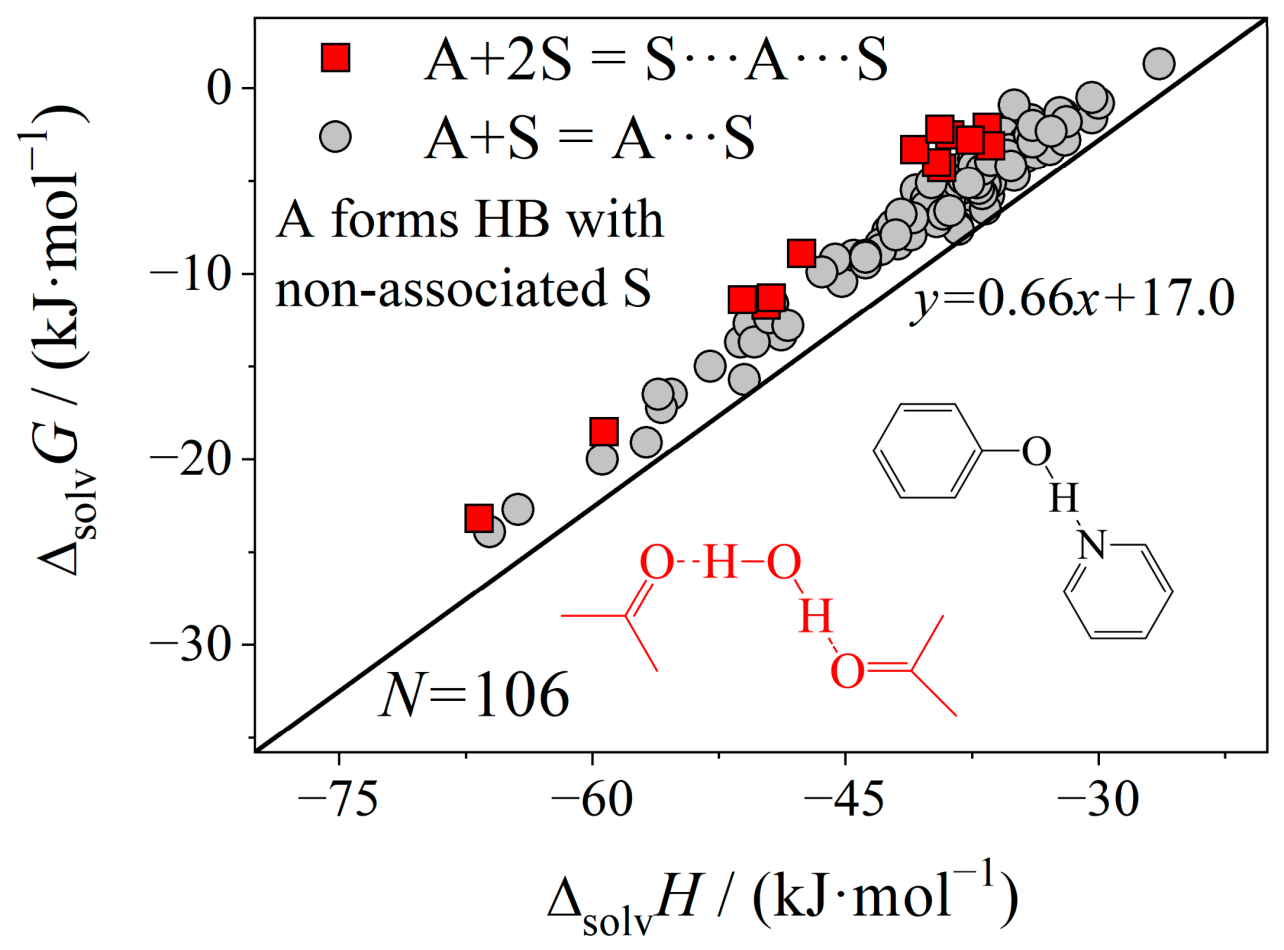
The graph compares the enthalpies and Gibbs energies of solvation accompanied by hydrogen bonding between a solute and a non-associated solvent. The structural formulas represent the examples of the systems considered. The line (*y* = 0.66*x* + 17.0) corresponds to the Barclay–Butler rule.

The graph comparison between the Δ_solv_*H* and Δ_solv_*G* in the systems exhibiting specific A-S interaction is provided in Figure 2. It contains the data previously compiled in Ref. [20] (66 data points), as well as the new Δ*G* vs. Δ*H* pairs calculated using the data from the recently published databases (see Table S2 for the numerical data) [25].

When a solute A is capable of hydrogen bonding with a solvent S, the specific interaction contribution (Δ_int(sp)_*H* and Δ_int(sp)_*G*) arises in the thermodynamic functions of solvation (Equation (4)). Thus, two types of interactions are present. The first one, associated with nondirectional bonding due to van der Waals interactions, might be called collective or nonspecific. The second, specific, or pairwise, interaction reflects complex formation due to directional bonding between A and S.

In Ref. [20], we showed that, in spite of the presence of the two intermolecular interaction types, the Δ*G* vs. Δ*H* relationship deviates from the Barclay–Butler line by a fairly constant value of 2.5 ± 0.7 kJ·mol^−1^, given that a 1:1 A···S complex is formed (e.g., ethanol in pyridine). Whenever 1:2 complexation happens (water in *N*,*N*-dimethylformamide), the deviation increases to ~5 kJ·mol^−1^. These deviations were initially found based on six Δ_solv_*G* vs. Δ_solv_*H* value pairs of proton donors in ethyl acetate and six Δ_solv_*G* vs. Δ_solv_*H* pairs of water in proton acceptors.

Thus, the following relation is valid for the solvation accompanied by A-S complexation:

(21) $$\Delta_{\mathrm{solv}}G^{A/S}=0.660\cdot \Delta_{\mathrm{solv}}H^{A/S}+\left(17.0+2.5\cdot n\right){\mathrm{kJ}\cdot \mathrm{mol}}^{-1}$$

where *n* is the number of directional bonds formed between A and S.

The deviation of 2.5*n* does not depend on the solute–solvent hydrogen bonding enthalpy [20], as long as the binding degree is close to one, which is usually the case upon solvation in the pure proton donor or acceptor media. Furthermore, it has an entropic origin. The 2.5*n* kJ·mol^−1^ term represents the Gibbs energy associated with an athermal complexation process, driven entirely by the entropic penalty of A···S binding in a solution. The entropy of athermal complexation can be described within a simplified statistical model, where a random sequence of A and S molecules (e.g., …AABB…) rearranges into a strictly paired arrangement (e.g., …ASAS…). For a system containing *N* distinguishable molecules of each species, the number of microstates decreases from Ω_init_ = (2*N*)! to Ω_fin_ = (*N*!)^2^·2*^N^*. This reduction yields an entropy change of Δ*S* = *k* ln (Ω_fin_/Ω_init_) ≈ −*R* ln 2 per mole of A-S mixture 1:1. Consequently, the Gibbs energy at 298.15 K is calculated as Δ*G* = –*T*Δ*S* ≈ 1.7 kJ·mol^−1^, which approximates the empirical value of 2.5 kJ·mol^−1^. This framework extends to solvation interactions, provided the mole-fraction scale is utilized. Increasing the coordination number of A in S raises the Ω_fin_/Ω_init_ ratio. For instance, in 1:2 complexation, Ω_init_ = (3*N*)!, Ω_fin_ = (2*N*)!*N*!, so Δ*G* ≈ *RT* ln (27/4) = 4.7 kJ·mol^−1^, agreeing with the observed deviation from the Barclay–Butler line of 5 kJ·mol^−1^ for water–proton acceptor systems [20]. The analogous considerations can be made for other coordination numbers, but such calculations may be difficult to verify against the measurements, as most molecular solvation and complexation data have been obtained for 1:1 and 1:2 binding.

It is worth noting that, when considering the solvation process in relatively weak proton acceptors, the 2.5*n* term may be influenced by the binding degree of A in S.

In Ref. [20], Equation (21) was checked against 58 (1:1) and eight (1:2) specifically interacting solute–solvent systems. In this work, we applied its rearranged form (Equation (22)) to calculate the solvation enthalpies in 40 solute–solvent systems from Δ_solv_*G*:

(22) $$\Delta_{\mathrm{solv}}H^{A/S}=\frac{\Delta_{\mathrm{solv}}G^{A/S}-\left(17.0+2.5\cdot n\right){\mathrm{kJ}\cdot \mathrm{mol}}^{-1}}{0.660}$$

**Figure 3:**
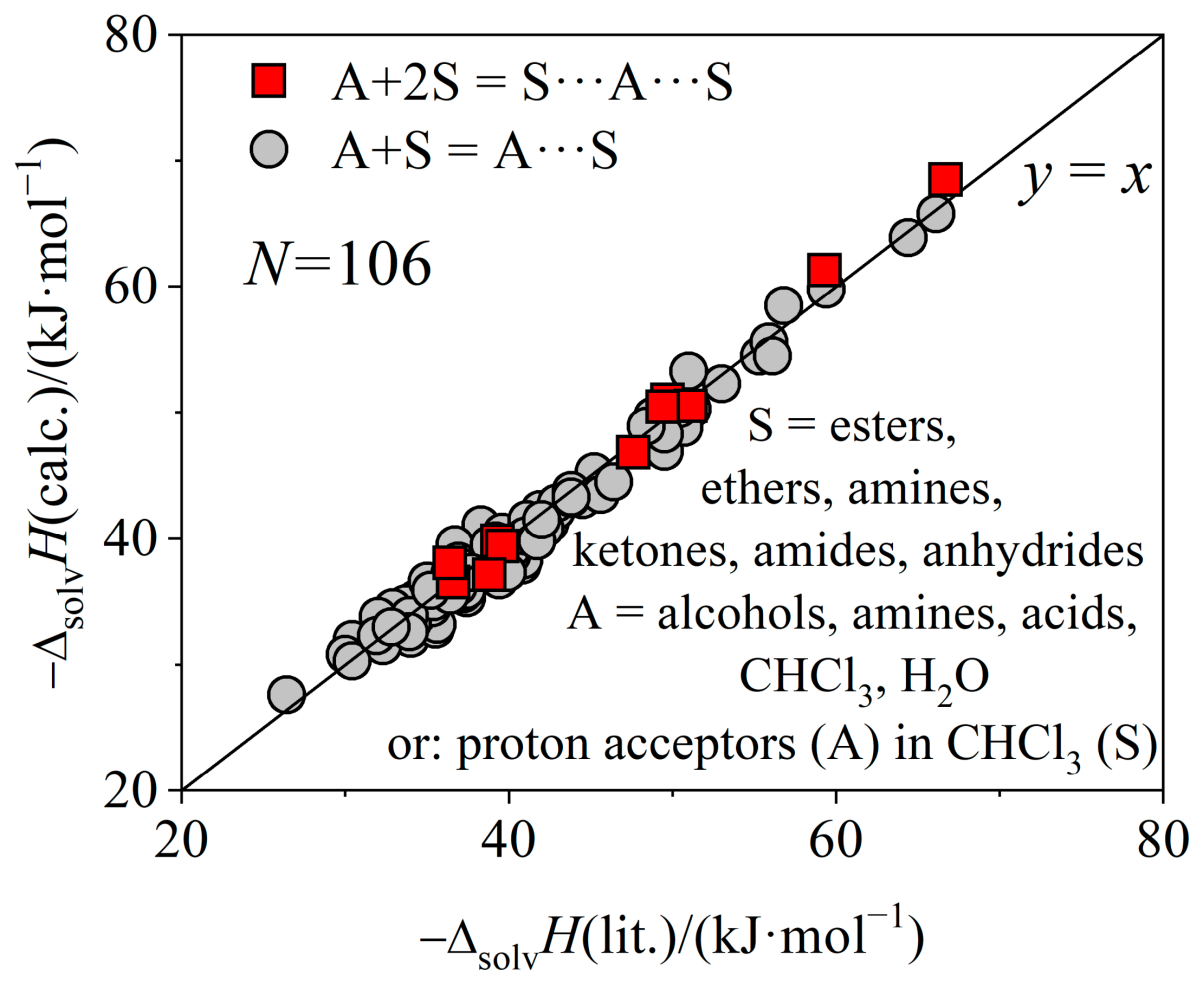
A comparison between the literature solvation enthalpies of organic non-electrolytes hydrogen-bonded to non-associated solvents and the values calculated from the solvation Gibbs energies at 298.15 K according to Equation (22).

We additionally analyzed 40 Δ_solv_*G* vs. Δ_solv_*H* pairs available from database [25]. The solutes considered included alcohols (methanol, ethanol, 1- and 2-propanol, 1-, 2-, tert- and isobutanol, 1-pentanol), water, acetic acid, and chloroform. The solvents were esters (dinonyl phthalate, propylene carbonate, butyl acetate, propyl acetate, methyl butanoate, propyl propanoate, isobutyl acetate), ethers (1-ethoxypropane, isopropyl ether, tetraglyme, *tert*-butyl ethyl ether, dimethoxymethane, tetrahydrofuran), ketones (cyclohexanone, 2-octanone, diphenylmethanone, 2-butanone, 4-methyl-2-pentanone, 3-methyl-2-butanone), amines (4-morpholine-carboxaldehyde, tri-*n*-pentylamine), amides (*N*,*N*-dimethylacetamide, *N*,*N*-dimethylformamide, 1-methyl-2-pyrrolidinone), and acetic anhydride. The numerical data are listed in Table S2. The comparison of the literature and calculated Δ_solv_*H* values (both from our previous work [20] and Table S2) is provided in Figure 3.

No systematic deviations were observed for a specific class of solutes or solvents. For 106 systems considered in total, *AD* and *rAD* were −0.2 kJ·mol^−1^ and −0.5%; *RMSD* and *rRMSD* were 1.3 kJ·mol^−1^ and 3.3%. *RMSD* was within the combined uncertainty of Equation (22) (analogous to Equations (19) and (20); we accept it as 1.5 kJ·mol^−1^); Δ_solv_*G* and Δ_solv_*H* were determined experimentally. For the 40 systems from Table S2, *AD* and *rAD* were −1.1 kJ·mol^−1^ and −2.8%; *RMSD* and *rRMSD* were 1.5 kJ·mol^−1^ and 3.8%.

#### 3.1.3. Solutes Incapable of Hydrogen Bonding Dissolved in Associated Solvents

**Figure 4:**
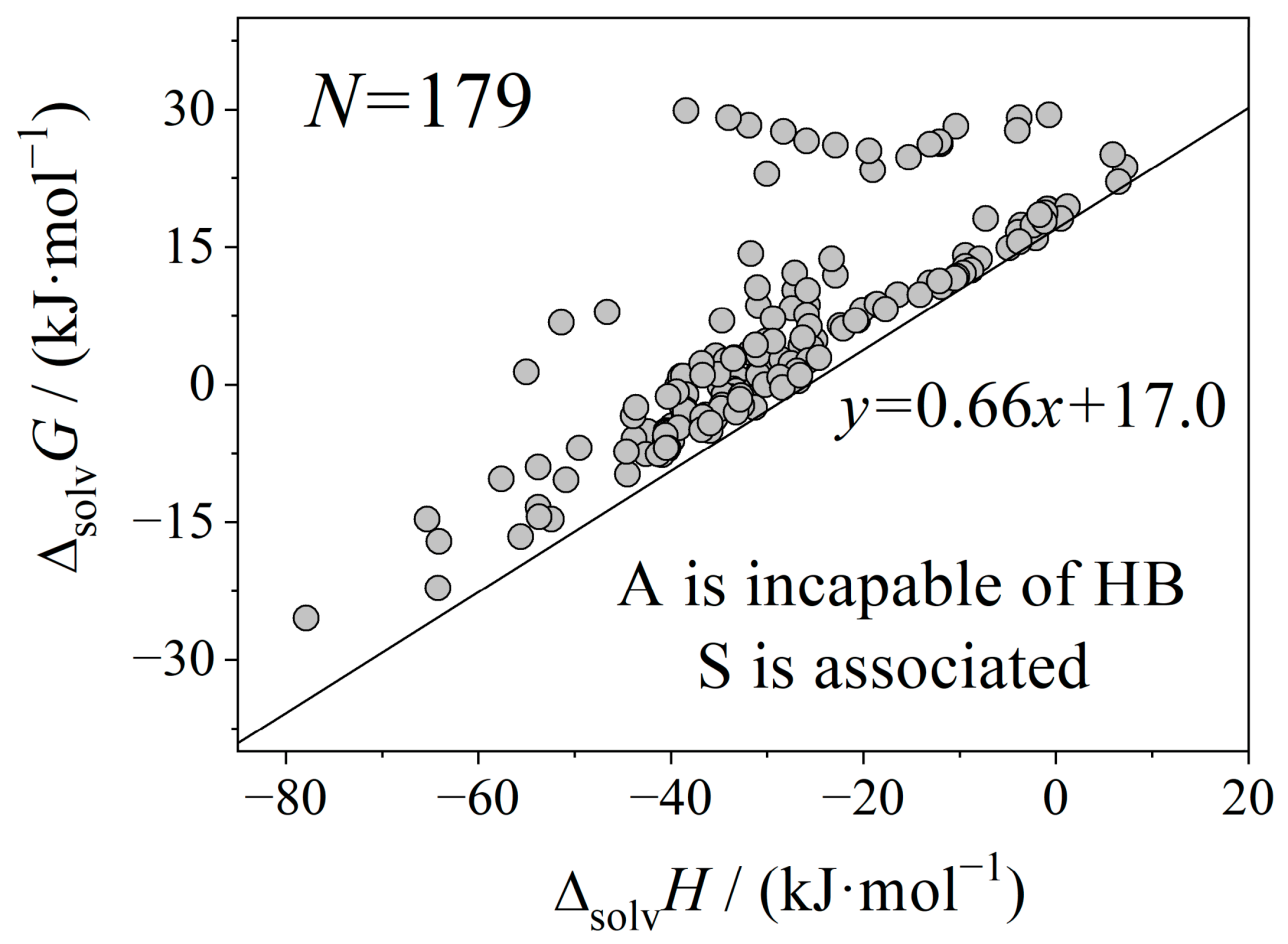
The graph comparing the enthalpies and Gibbs energies of solvation of organic non-electrolytes in associated solvents. The line (*y* = 0.66*x* + 17.0) corresponds to the Barclay–Butler rule.

In Figure 4, Δ_solv_*G* and Δ_solv_*H* of 179 organic non-electrolytes incapable of hydrogen bonding in a series of associated solvents are compared. In red, the Barclay–Butler line (Equation (17)) is depicted. The numerical data are listed in Table S4 (54 values) and additionally taken from Ref. [20] (125 values).

In the previous studies [67,68,69,70], it was shown that the deviations from the Barclay–Butler line towards more positive Gibbs energies of solvation are due to the solvophobic effect in associated solvents. Its contribution to the thermodynamic functions of solvation is given by Equations (23) and (24):

Δ_solv_*G* = Δ_solv(nonsp)_*G* + Δ_s.e._*G* (23)

Δ_solv_*H* = Δ_solv(nonsp)_*H* + Δ_s.e._*H* (24)

Thus, the systems in Figure 4 obey Equation (25):

(25) $$\Delta_{\mathrm{solv}}G^{A/S}=17.0{\;\mathrm{kJ}\cdot \mathrm{mol}}^{-1}+\Delta_{s.e.}G^{A/S}+0.660\cdot \left(\Delta_{\mathrm{solv}}H^{A/S}-\Delta_{s.e.}H^{A/S}\right)$$

In works [67,68,69,70,71], the enthalpic contribution Δ_s.e._*H* was shown to be within the experimental uncertainty for the majority of associated solvents. The exceptions were (a) water and (b) formamide; in the latter case, only six aromatic molecules exhibited an enthalpic solvophobic effect [72]. Thus, in the vast majority of cases, the deviation from the Barclay–Butler line was supposed to be due to Δ_s.e._*G*. The latter was found to obey a linear correlation (Equation (26)) with a characteristic volume (*V*_x_) [73] of a solvent molecule [68,74]:

Δ_s.e._*G* = *k_S_*·*V*_x_^A^ + *b*_S_ (26)

where *k_S_* and *b_S_* are the coefficients specific to each solvent (see Table S3). It is important to mention that Equation (26) was initially derived using the independent approaches for the extraction of the Gibbs energies and enthalpies of non-specific interaction, with no assumptions regarding the Δ_solv_*H −* Δ_solv_*G* connection [68,74].

In the absence of an enthalpic contribution to the solvophobic effect, Equation (25) can be rearranged to calculate the solvation enthalpy from the solvation Gibbs energy:

(27) $$\Delta_{\mathrm{solv}}H^{A/S}=\frac{\Delta_{\mathrm{solv}}G^{A/S}-\Delta_{s.e.}G^{A/S}-17.0{\;\mathrm{kJ}\cdot \mathrm{mol}}^{-1}}{0.660}$$

**Figure 5:**
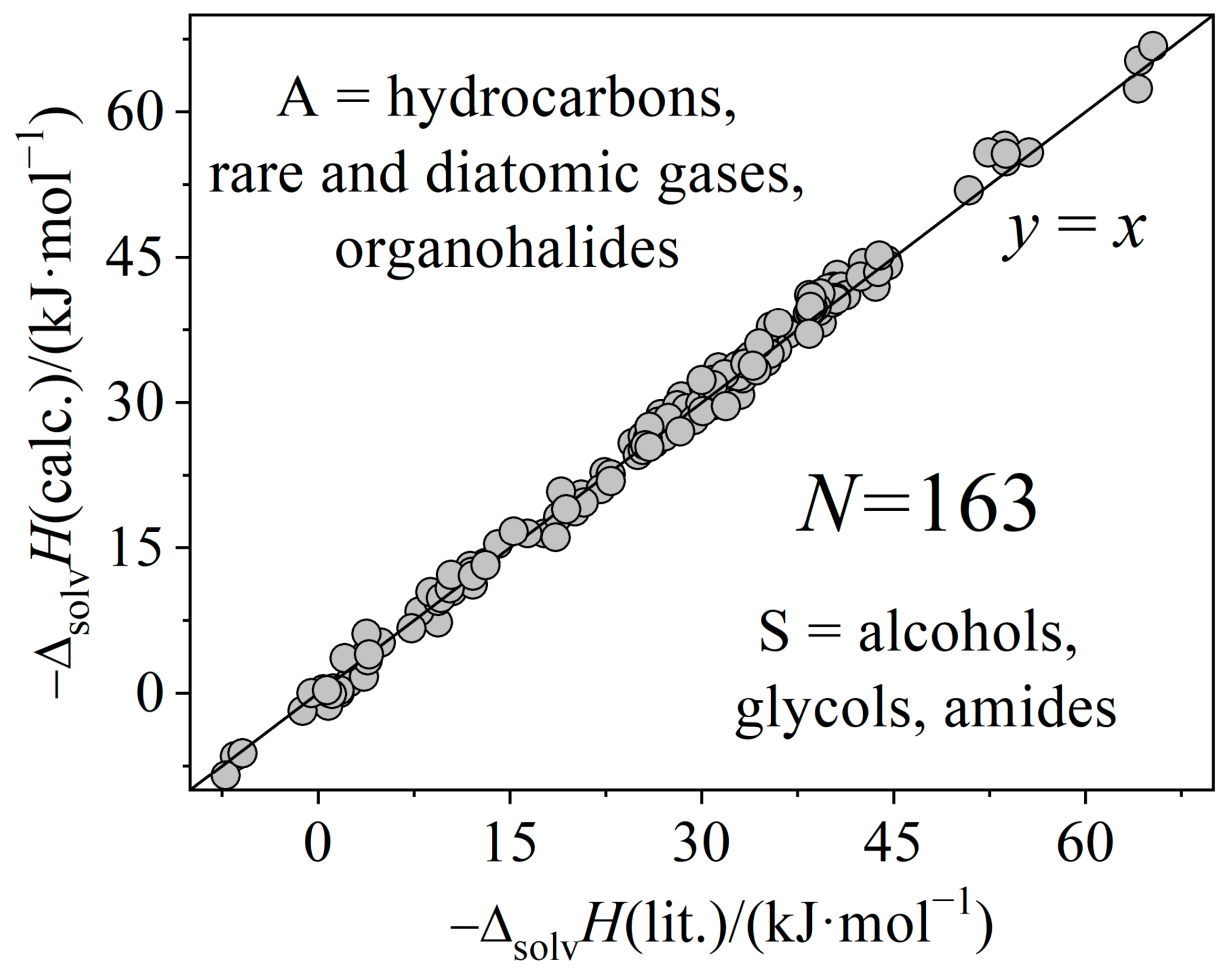
A comparison of Δ_solv_*H* of aliphatic and aromatic derivatives in associated solvents calculated using the data on Δ_solv_*G* and Δ_s.e._*G* according to Equation (27) with the literature.

In Figure 5, Δ_solv_*H* of aliphatic and aromatic derivatives in associated solvents, calculated using the data on Δ_solv_*G* and Δ_s.e._*G* according to Equation (27), are compared with the literature. These include the values from our previous work [20] and those from Refs. [68,70,75], listed in Table S4. The solutes included hydrocarbons, their halogenated derivatives, rare and diatomic gases, while the solvents were alcohols, glycols, and amides.

For 163 systems considered, *AD* and *rAD* were 0.4 kJ·mol^−1^ and 1.2%; *RMSD* and *rRMSD* were 1.2 kJ·mol^−1^ and 4.4%, which are within the expected errors. For the 55 enthalpy values collected in Table S4, *AD* and *rAD* were −1.0 kJ·mol^−1^ and 3.0%; *RMSD* and *rRMSD* were 1.3 kJ·mol^−1^ and 3.8%.

Thus, except for water, the deviations of the (Δ_solv_*H* vs. Δ_solv_*G*) pairs in associated solvents from the Barclay–Butler line are due to the entropic contribution. The reasons for the peculiar behavior of water are currently unclear to us.

#### 3.1.4. Solutes Hydrogen-Bonded to Associated Solvents

**Figure 6:**
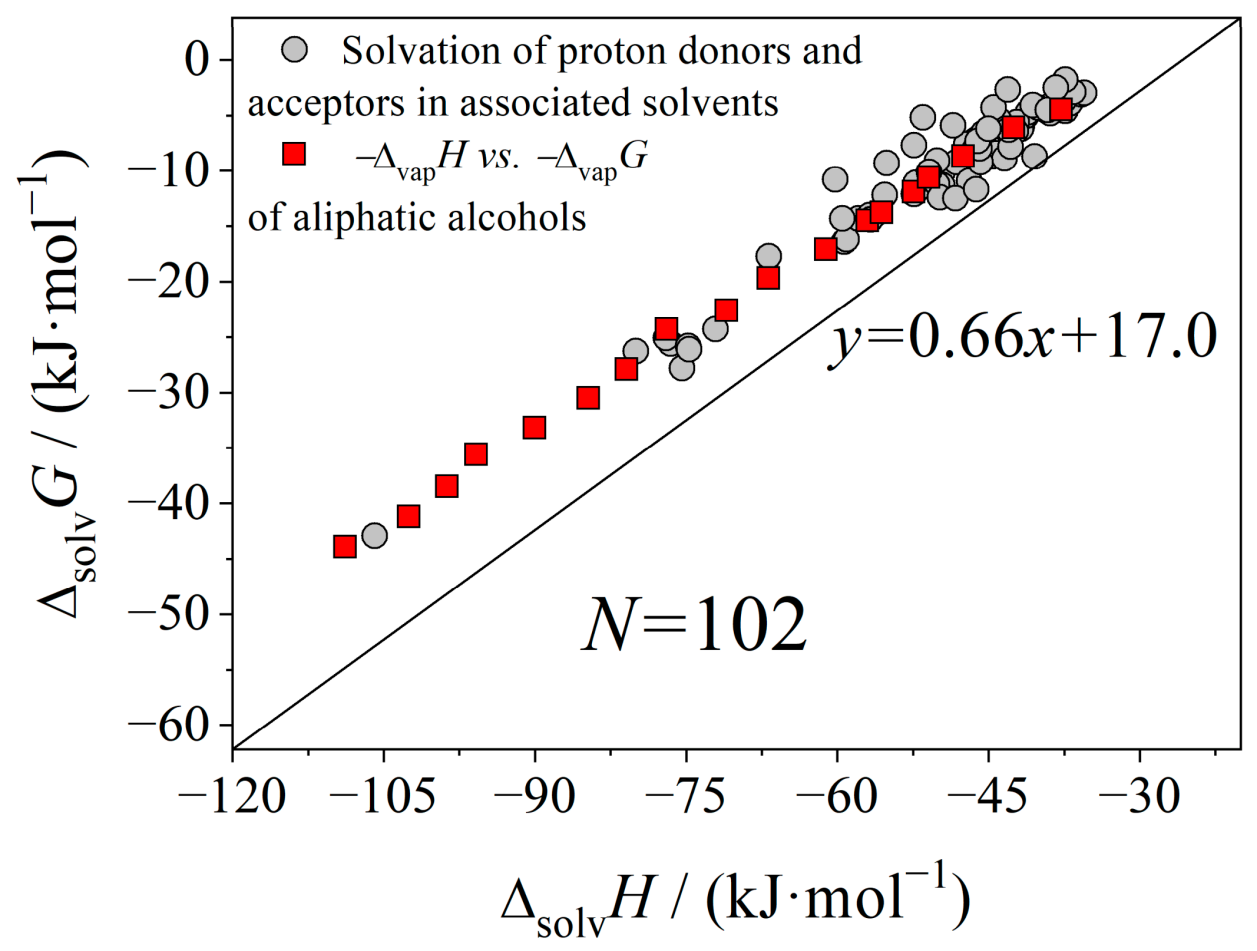
The graph compares the enthalpies and Gibbs energies of organic non-electrolytes solvation in associated solvents accompanied by solute–solvent hydrogen bonding. The line (*y* = 0.66*x* + 17.0) corresponds to the Barclay–Butler rule.

At the qualitative level, the Δ_solv_*G* vs. Δ_solv_*H* relationship when the solute is capable of hydrogen bonding with an associated solvent is similar to what is presented in Section 3.1.4 (see Figure 6). Δ_s.e._*G* contribution, depending on the medium solvophobicity and solute *V*_x_, remains, with an added specific interaction contribution.

However, the character of hydrogen bonding between a solute (A) and an associated solvent (S) depends on features of S-S hydrogen bonding. An associated solvent is an equilibrium mixture of monomers and oligomers. A solute molecule, a proton donor or/and acceptor, competes with a solvent for an active hydrogen atom or lone electron pair in S molecules. It influences the thermodynamic functions of solvation. In a series of works devoted to the thermodynamics of proton donors/acceptors’ solvation in associated solvents, a rule concerning the solute–solvent competition character was proposed [76]. The presence of such a competition depends on (a) the ratio between the number of lone pairs and active protons in the solvent molecule and (b) whether the solute is a proton donor or acceptor. For example, a methanol molecule possesses two lone pairs and an active proton. The association of methanol as a solvent is due to a single lone pair, while the remainder is free. For proton-donating solutes, there will be no competition with a solvent, so methanol will behave as a non-associated ether. In the case of a proton-accepting solute, competition with a solvent would be inevitable. In solvents with lone pairs − active proton ratio 1:1 (water, formamide), the competition will be present regardless of the proton-donating/accepting ability of the solute. Thus, we will separately discuss the two cases leading to different Δ_solv_*G* vs. Δ_solv_*H* relations:

1. Non-competitive solvent–solute hydrogen bonding.
2. Competitive solvent–solute hydrogen bonding.

##### Noncompetitive Solvent–Solute Hydrogen Bonding

In the case of noncompetitive hydrogen bonding between a solute and associated solvent, each contribution to the thermodynamic functions of solvation (Δ_solv(nonsp)._*f*, Δ_int(sp)._*f*, Δ_s.e._*f*) is added directly, so the Δ_solv_*G* vs. Δ_solv_*H* relationship takes the following form:

(28) $$\Delta_{\mathrm{solv}}G^{A/S}=\left(17.0+2.5\cdot n\right){\mathrm{kJ}\cdot \mathrm{mol}}^{-1}+\Delta_{s.e.}G^{A/S}+0.660\cdot \Delta_{\mathrm{solv}}H^{A/S}$$

We used this relation in its rearranged form to calculate the solvation enthalpies and compare them with the literature:

(29) $$\Delta_{\mathrm{solv}}H^{A/S}=\frac{\Delta_{\mathrm{solv}}G^{A/S}-\Delta_{s.e.}G^{A/S}-\left(17.0+2.5\cdot n\right){\mathrm{kJ}\cdot \mathrm{mol}}^{-1}}{0.660}$$

**Figure 7:**
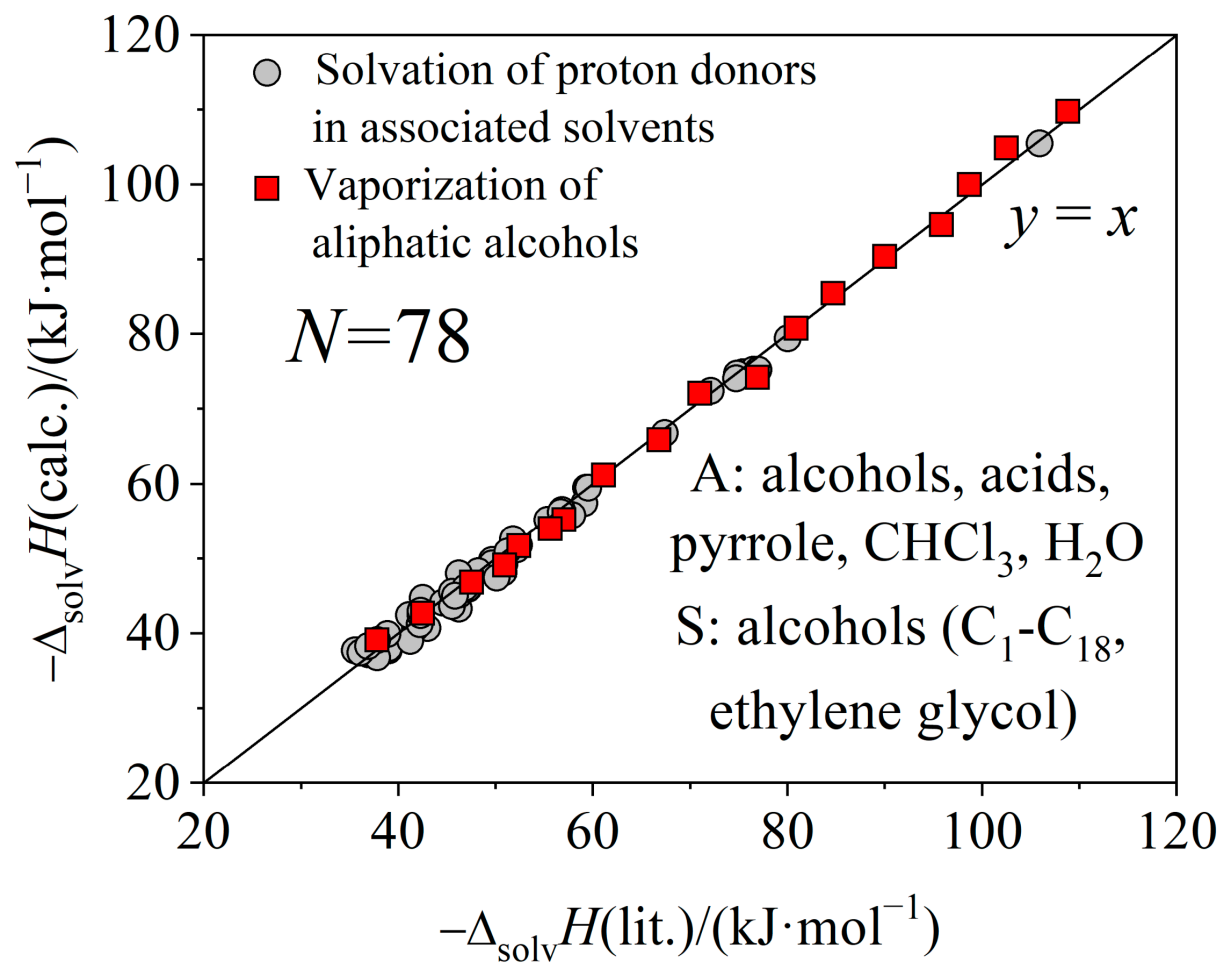
A comparison of Δ_solv_*H* of proton donors in alcohols calculated using the data on Δ_solv_*G* and Δ_s.e._*G* according to Equation (29) with the literature.

The comparison is shown in Figure 7. The data include the values earlier presented in Ref. [20] and Δ_solv_*G* and Δ_solv_*H*, found using the data from Refs. [25,77,78,79]. The latter magnitudes, together with the auxiliary Δ_s.e._*G* and calculated Δ_solv_*H* values, are listed in Table S5.

The systems shown in Figure 7 include various proton donors (alcohols, acids, pyrrole, chloroform, water) dissolved in alcohols from methanol to 1-octadecanol, as well as ethylene glycol. The data on 78 systems were compared. *AD* and *rAD* were −0.4 kJ·mol^−1^ and −0.7%; *RMSD* and *rRMSD* were 1.3 kJ·mol^−1^ and 2.3%. For the 50 enthalpies collected in Table S5, *AD* and *rAD* were −0.9 kJ·mol^−1^ and 1.8%; *RMSD* and *rRMSD* were 1.2 kJ·mol^−1^ and 2.4%.

##### Competitive Solvent–Solute Hydrogen Bonding

Competitive hydrogen bonding can be considered as a substitution reaction:

(30) $$A_{\left(S\right)}+S_{n(S)}⇄A\cdots S_{m(S)}+S_{n\text{-}m(S)}$$

For example, Equation (30) applies to proton acceptor solutions in alcohols. Whether A = proton acceptor or donor, Equation (30) applies to solutions in water or formamide since there are equal lone pair and active proton counts in these molecules (2).

Equation (30) can be decomposed into the two stages, Equations (31) and (32):

(31) $$S_{n(S)}⇄S_{m(S)}+S_{n\text{-}m(S)}$$

(32) $$A_{\left(S\right)}+S_{m(S)}⇄A\cdots S_{m(S)}$$

Each stage contributes to the total thermodynamic functions of solvation. The 2.5*n* term, arising in Equations (21) and (28) due to specific interactions, cancels out for the processes of S_n_ dissociation and A-S association when A-S binding is complete (α^A/S^ ≈ 0). Thus, the Δ_solv_*G* vs. Δ_solv_*H* relationship can be written as follows, as long Δ_s.e._*H* is absent:

(33) $$\Delta_{\mathrm{solv}}G^{A/S}=17.0{\;\mathrm{kJ}\cdot \mathrm{mol}}^{-1}+\Delta_{s.e.}G^{A/S}+0.660\cdot \Delta_{\mathrm{solv}}H^{A/S}$$

Incomplete binding of A in S slightly decreases the intercept of Equation (33) [80]. The solvation enthalpy can be calculated via Equation (34):

(34) $$\Delta_{\mathrm{solv}}H^{A/S}=\frac{\Delta_{\mathrm{solv}}G^{A/S}-\Delta_{s.e.}G^{A/S}-17.0{\;\mathrm{kJ}\cdot \mathrm{mol}}^{-1}}{0.660}$$

**Figure 8:**
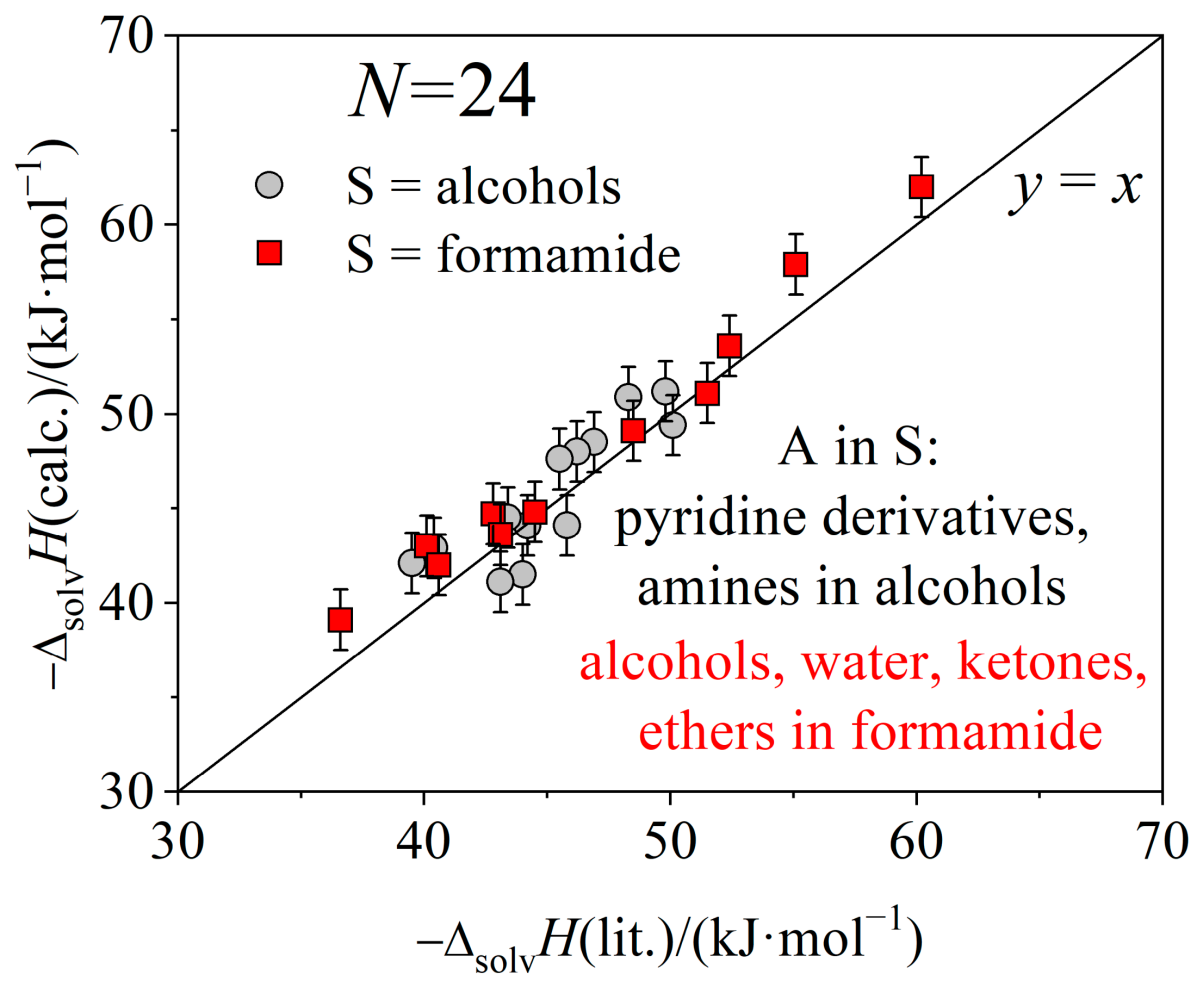
A comparison of Δ_solv_*H* of proton acceptors in alcohols and proton donors in formamide calculated using the data on Δ_solv_*G* and Δ_s.e._*G* according to Equation (34) with the literature.

In Table S6, the literature Gibbs energies and enthalpies of solvation of proton acceptors in aliphatic alcohols [80] and those of proton donors/acceptors in formamide [81,82] are listed together with the Δ_solv_*H* values calculated according to Equation (34). For these systems, the binding degree is expected to be one, so Equation (34) is valid. In Ref. [72], Δ_s.e._*H* of alkanes in formamide was shown to be nearly zero; the same approximation was accepted for the solutions in formamide listed in Table S7. The graph comparison is given in Figure 8. *AD* and *rAD* were 1.0 kJ·mol^−1^ and 2.1%; *RMSD* and *rRMSD* were 1.9 kJ·mol^−1^ and 4.0%.

Summarizing, the above classification covers most possible solute–solvent systems with respect to hydrogen bonding capability, except for the systems with intramolecular hydrogen bonds. The systematic analysis of solvation thermodynamics for more than 1000 experimental Δ_solv_*G* vs. Δ_solv_*H* pairs establishes that the relationships between these quantities are governed by a small set of physically meaningful contributions, namely the constant entropic penalty per directional bond formation, solvophobic effects, and conformational flexibility in long-chain aliphatics. Quantitative understanding of the origin of each deviation sheds new light on a Gibbs energy equation, enabling the use of the Δ_solv_*G* vs. Δ_solv_*H* relationship as a powerful predictive tool.

Furthermore, below we show that it has a number of outcomes applied to complexation thermodynamics.

### 3.2. The Relationships Between the Gibbs Energies, Enthalpies, and Entropies of Molecular Complexation

Relationships between Δ_bind_*G*, Δ_bind_*S*, and Δ_bind_*H* of molecular compounds, mostly in solutions, have gained attention since the 1950s. Leffler [83] highlighted the presence of correlations between Δ*S* and Δ*H* of amines–trimethylboron complex dissociation, along with ionization of several acids and bases in solutions and isokinetic relationships. Pimentel and McClellan [84,85] showed that there are monotonic Δ_bind_*S* vs. Δ_bind_*H* relationships in a series of processes where a given proton donor binds with a certain type of proton acceptor in an inert solvent. More correlations have been found later [29,86,87]. Still, the analysis and distinction of the experimental data on binding thermodynamics alone may sometimes be complicated due to demanding measurement procedures and accumulated errors, as discussed in Section 2.4.

Below we demonstrate that transformations of the equations connecting Δ_solv_*G* and Δ_solv_*H*, which were derived above based on the fairly precise experimental data, enable obtaining the single linear relationship between Δ_bind_*G* and Δ_bind_*H*, applicable both to hydrogen bonding and charge-transfer complexation in solution. Furthermore, its transformation leads to a Δ_bind(g)_*G* vs. Δ_bind(g)_*H* relationship. These relations appear to cover the molecular complexes of diverse structures in a wide range of binding enthalpies and Gibbs energies.

#### 3.2.1. The Relationships Between Δ*G*, Δ*H*, and Δ*S* of Molecular Complexation in Solution

The framework for the thermodynamic description of the complexation process is presented in Section 2.3. In the absence of hydrogen bonding, Δ_solv_*G* and Δ_solv_*H* are entirely defined by nonspecific interactions, i.e., Equation (17) can be rewritten as follows:

(35) $$\Delta_{\mathrm{solv}(\mathrm{nonsp})}G^{A/S}/\left({\mathrm{kJ}\cdot \mathrm{mol}}^{-1}\right)=17.0+0.660\cdot \Delta_{\mathrm{solv}(\mathrm{nonsp})}H^{A/S}/\left({\mathrm{kJ}\cdot \mathrm{mol}}^{-1}\right)$$

When the solvation is accompanied by hydrogen bonding between a solute and non-associated solvent, Δ_solv_*G* and Δ_solv_*H* are governed by Equation (21) with the same slope and an intercept of (17.0 + 2.5*n*) kJ·mol^−1^. By subtracting Equation (35) from Equation (21), one arrives at the relationship between the Gibbs energies and enthalpies of specific interaction [20]:

(36) $$\Delta_{\mathrm{int}(\mathrm{sp})}G^{A/S}/\left({\mathrm{kJ}\cdot \mathrm{mol}}^{-1}\right)=2.5n+0.660\times \Delta_{\mathrm{int}(\mathrm{sp})}H^{A/S}/\left({\mathrm{kJ}\cdot \mathrm{mol}}^{-1}\right)$$

It follows from Equations (8)–(10) that, at non-bound solute fraction *α*^A/S^ ≈ 0, Δ_int(sp)_*G*^A/S^ ≈ Δ_bind(S)_*G*^A···S^ and Δ_int(sp)_*H*^A/S^ ≈ Δ_bind(S)_*H*^A···S^. Furthermore, since the 1970s [88,89,90,91], it has been repeatedly highlighted that the thermodynamic functions of formation of a strong A···B complex in a B solution are nearly equal to those in an inert solvent (primarily tetrachloromethane):

Δ_int(sp)_*f*^A/B^ ≈ Δ_bind(B)_*f*^A···B^ ≈ Δ_bind(S)_*f*^A···B^ (37)

Thus, Equation (36) transforms to the connection between the Gibbs energies and enthalpies of complex formation in an inert solvent (Equation (38)):

(38) $$\Delta_{\mathrm{bind}(S)}G^{A\cdots B}/\left({\mathrm{kJ}\cdot \mathrm{mol}}^{-1}\right)=2.5n+0.660\times \Delta_{\mathrm{bind}(S)}H^{A\cdots B}/\left({\mathrm{kJ}\cdot \mathrm{mol}}^{-1}\right)$$

In the rearranged form, Equation (38) enables the calculation of the binding enthalpy from the Gibbs energy at 298.15 K (i.e., the data on equilibrium at a single temperature) (Equation (39)):

(39) $$\Delta_{\mathrm{bind}(S)}H^{A\cdots B}=\frac{\Delta_{\mathrm{bind}(S)}G^{A\cdots B}-2.5n{\;\mathrm{kJ}\cdot \mathrm{mol}}^{-1}}{0.660}$$

In Ref. [21], we demonstrated that Equation (39) applies to 866 hydrogen-bonded and charge-transfer complexes of diverse chemical structures. In total, 647 HB complexes (306 proton acceptors and 60 proton donors) and 219 CT complexes (six electron acceptors and 180 electron donors) were considered. Proton donors included substituted phenols, alkanols, carboxylic acids, NH- and CH-acids, and thiols. Electron acceptors were iodine, bromine, iodine bromide, iodine chloride, tetracyanoethylene, and chloranil. Proton acceptors, largely overlapping with electron donors, included unsaturated and aromatic hydrocarbons, ethers, carbonyl-containing compounds, saturated halohydrocarbons, nitriles, nitrogen heterocycles, amines, amides and thioamides, organosulfur, organoselenium, and organophosphorus compounds. The *RMSD* between the experimental enthalpies and the values calculated from Δ_bind(S)_*G*^A···B^ according to Equation (39) was 1.5 kJ·mol^−1^.

Most HB and CT complexes are characterized by Δ_bind(S)_*H*^A···B^ of 10–30 kJ·mol^−1^. It is then not surprising that multiple enthalpy–entropy (or enthalpy–Gibbs energy) correlations could be proposed before, satisfactorily describing the experimental data. However, it is important to note that Equation (39) was applied to far stronger complexes, e.g., dichloroacetic acid–diphenyl selenoxide, iodine–tetramethylthiourea adducts with Δ_bind(S)_*H*^A···B^ of 50–60 kJ·mol^−1^ [21]. In this work, we attempted to find more data on strongly bound complexes and considered homodimerization due to hydrogen bonding. In Table 3, Δ*G* and Δ*H* of dimerization of carboxylic acids in benzene, lactams, and phenols in tetrachloromethane are presented.

The calculated and literature Δ_bind(S)_*H* agree within 2 kJ·mol^−1^ for all the dimers in Table 3, except *β*-lactam. The deviation of 4.0 kJ·mol^−1^ in the case of *β*-lactam can hardly be attributed to peculiarities of the molecular structure and is close to the combined uncertainty of experimental Δ_bind(S)_*H* (Section 2.4) and Equation (39).

Taking into account the wide applicability, together with the fact that Equation (39) was derived based on independent considerations in solvation thermodynamics (i.e., it is not based on experimental data on complexation thermodynamics), we believe that the linear relations (38) and (39) might represent a rule. In our opinion, it has several important outcomes. First, this is a tool for straightforward estimation of the binding enthalpy and the complexation constant temperature dependence from a single *K*_bind_ measurement at 298.15 K (or another temperature). Second, in Ref. [94], we showed that, together with the well-known Abraham’s equation (log *K*_HB,c_ = 7.354α^H^β^H^ − 1.094), Equation (39) can be used for the determination of the binding enthalpies directly from the molecular acidity and basicity descriptors α^H^ and β^H^. The current data accumulated on α^H^ and β^H^ enable the estimation of Δ_bind(S)_*H* in more than 3.6·10^5^ donor–acceptor systems according to Equation (40):

Δ_bind(S)_*H*/(kJ·mol^−1^) = −63.61α^H^β^H^ + 3.03 (40)

Third, the linear relations (38) and (39) demonstrate that proton donor (electron acceptor) and proton acceptor (electron donor) abilities scale based on the enthalpies and that the complex formation constants are equivalent.

#### 3.2.2. The Relationships Between Δ*G*, Δ*H*, and Δ*S* of Gas-Phase Molecular Complexation

Less experimental data are available on the thermodynamics of gas-phase molecular complexation. Nonetheless, gas-phase binding has been an important model process for the analysis of the enthalpy–entropy compensation phenomenon based on the estimated/computed Δ_bind(g)_*S* and Δ_bind(g)_*H* values [10,11,95]. We showed that the results obtained in Section 3.1.1, Section 3.1.2 and Section 3.2.1 necessarily lead to the linear Δ_bind(g)_*G* vs. Δ_bind(g)_*H* relationship with the same slope of 0.660 and checked its validity against both computed and experimental data [22].

The connections between the thermodynamic parameters of solvation and binding in different phases can be illustrated as follows:

(41) $$\begin{array}{c} {\;A}_{\left(g\right)}+B_{\left(g\right)}\overset{\Delta_{\mathrm{bind}(g)}f^{A\cdots B}}{\to }A_{\left(g\right)}\cdots B_{\left(g\right)}\\ \Delta_{\mathrm{solv}}f^{A/S}↓\;↓\Delta_{\mathrm{solv}}f^{B/S}\;↓\Delta_{\mathrm{solv}}f^{A\cdots B/S}\;\\ {\;A}_{\left(S\right)}+B_{\left(S\right)}\underset{\Delta_{\mathrm{bind}(S)}f^{A\cdots B}}{\to }A_{\left(S\right)}\cdots B_{\left(S\right)} \end{array}$$

From the thermodynamic cycle (41), the difference between the thermodynamic parameters of solution- and gas-phase complexation is equal to the difference between the solvation parameters of the complex and its constituents (Equation (42)):

(42) $$\begin{array}{c} \Delta_{\mathrm{bind}(S)}f^{A\cdots B}-\Delta_{\mathrm{bind}(g)}f^{A\cdots B}=\\ \Delta_{\mathrm{solv}}f^{A\cdot \cdot \cdot B/S}-\Delta_{\mathrm{solv}}f^{A/S}-\Delta_{\mathrm{solv}}f^{B/S} \end{array}$$

The relationship between the Gibbs energies and enthalpies of complex formation in an inert solvent is described by Equation (38). Both monomers and their complex exhibit nonspecific interactions with an inert solvent, so the thermodynamic parameters of their solvation are connected via Equation (17). The solvation process corresponds to the transfer from an ideal gas to an infinitely diluted solution. Then Δ_bind(g)_*G*^A···B^ can be expressed through Δ_bind(S)_*G*^A···B^ and Δ_solv_*G* of A, B, and A···B. Combining Equations (17), (38) and (42), one comes to Equation (43):

(43) $$\begin{array}{c} \Delta_{\mathrm{bind}(g)}G^{A\cdots B}/\left({\mathrm{kJ}\cdot \mathrm{mol}}^{-1}\right)=0.660\times \Delta_{\mathrm{bind}(S)}H^{A\cdots B}/\left({\mathrm{kJ}\cdot \mathrm{mol}}^{-1}\right)+2.5n-\\ -0.660\times \Delta_{\mathrm{solv}}H^{A\cdots B/S}/\left({\mathrm{kJ}\cdot \mathrm{mol}}^{-1}\right)-17.0+\\ +0.660\times \Delta_{\mathrm{solv}}H^{A/S}/\left({\mathrm{kJ}\cdot \mathrm{mol}}^{-1}\right)+17.0+\\ +0.660\times \Delta_{\mathrm{solv}}H^{B/S}/\left({\mathrm{kJ}\cdot \mathrm{mol}}^{-1}\right)+17.0 \end{array}$$

The difference Δ_bind(S)_*H*^A···B^ − Δ_solv_*H*^A···B/S^ + Δ_solv_*H*^A/S^ + Δ_solv_*H*^B/S^ equals Δ_bind(g)_*H*^A···B^ (Equation (42)). Then, the relationship between the Gibbs energies and enthalpies of gas-phase complexation (Equation (44)) can be obtained [22]. Its slope is the same as for the solvation and solution-phase complexation processes.

(44) $$\Delta_{\mathrm{bind}(g)}G^{A\cdots B}/\left({\mathrm{kJ}\cdot \mathrm{mol}}^{-1}\right)=17.0+2.5n+0.660\times \Delta_{\mathrm{bind}(g)}H^{A\cdots B}/\left({\mathrm{kJ}\cdot \mathrm{mol}}^{-1}\right)$$

Conversely, Δ_bind(g)_*H*^A···B^ can be found according to Equation (45):

(45) $$\Delta_{\mathrm{bind}(g)}H^{A\cdots B}/\left({\mathrm{kJ}\cdot \mathrm{mol}}^{-1}\right)=\frac{\Delta_{\mathrm{bind}(g)}G^{A\cdots B}/\left({\mathrm{kJ}\cdot \mathrm{mol}}^{-1}\right)-17.0-2.5n}{0.660}$$

In Table 4, the comparison is made between some experimental Δ_bind(g)_*H*^A···B^ available from the literature and the values calculated from experimental Δ_bind(g)_*G*^A···B^ via Equation (45).

The enthalpy measurements, as they require tracking of the temperature dependence of the binding constant, are relatively rare. On the other hand, the modern computational approaches [106,107] enable fairly accurate estimation of Δ_bind(g)_*H*. At the same time, the rigid rotor–harmonic oscillator approximation commonly employed in thermodynamic function calculation contributes significantly to the uncertainties of Δ_bind(g)_*S* and Δ_bind(g)_*G*. In Ref. [22], we extended the available data on the binding thermodynamics by computing the Δ_bind(g)_*H* values for the 67 compounds whose Δ_bind(g)_*G* were available from the literature. The coupled-cluster method with complete basis set extrapolation was used to attain satisfactory accuracy. The computed electronic binding energies were compared to the literature values obtained using the same or higher level of theory for 13 systems, and the additional benchmark computations at the higher theory level were performed for 19 systems composed of relatively small molecules to ensure the accuracy [22].

In total, 63 out of 67 systems obeyed Equation (45) within the limits of combined uncertainty of 4.7 kJ·mol^−1^. The latter includes the uncertainties of Equations (17) and (38) underlying Equation (45) (3.6 kJ·mol^−1^) and the expected errors of Δ_bind(g)_*G*(lit) and Δ_bind(g)_*H*(QC) (3 kJ·mol^−1^). Less data were available on gas-phase binding, compared to the solution-phase complexation and solvation. Nonetheless, the systems included quite diverse donors and acceptors. Hydrogen-bonded complexes were formed by alcohols, acids, acetylene, sulfur dioxide, chloroform, amines, ethers, ketones and aldehydes, pyridine, trimethylphosphine, and alkenes. There were also charge-transfer complexes between iodine, tetracyanoethylene, on the one hand, and ethers, sulfides, aromatics, on the other hand. In addition, acetone, acetaldehyde, and chloromethane dimers, which are mostly contributed by dispersion interactions, were considered. The four outliers did not possess specific structural features.

Thus, the comparison between the computed binding enthalpies and the values calculated from the equilibrium constants at 298.15 K confirmed the validity of Equation (45) and its applicability to the wide range of gas-phase molecular complexes, regardless of their nature and interaction strength. Equations (44) and (45) follow from the fundamental laws of thermodynamics (additivity of state functions) applied to the solvation and solution-phase complexation, checked against more than 2000 Δ*G* vs. Δ*H* pairs in total [19,20,21]. Then it is reasonable to call Equations (44) and (45) the rule as well.

### 3.3. Summary on Solvation/Molecular Complexation Processes and Further Implications in Macromolecular Recognition

#### 3.3.1. Gibbs Equation Applied to Solvation and Molecular Complexation: A Summary

**Figure 9:**
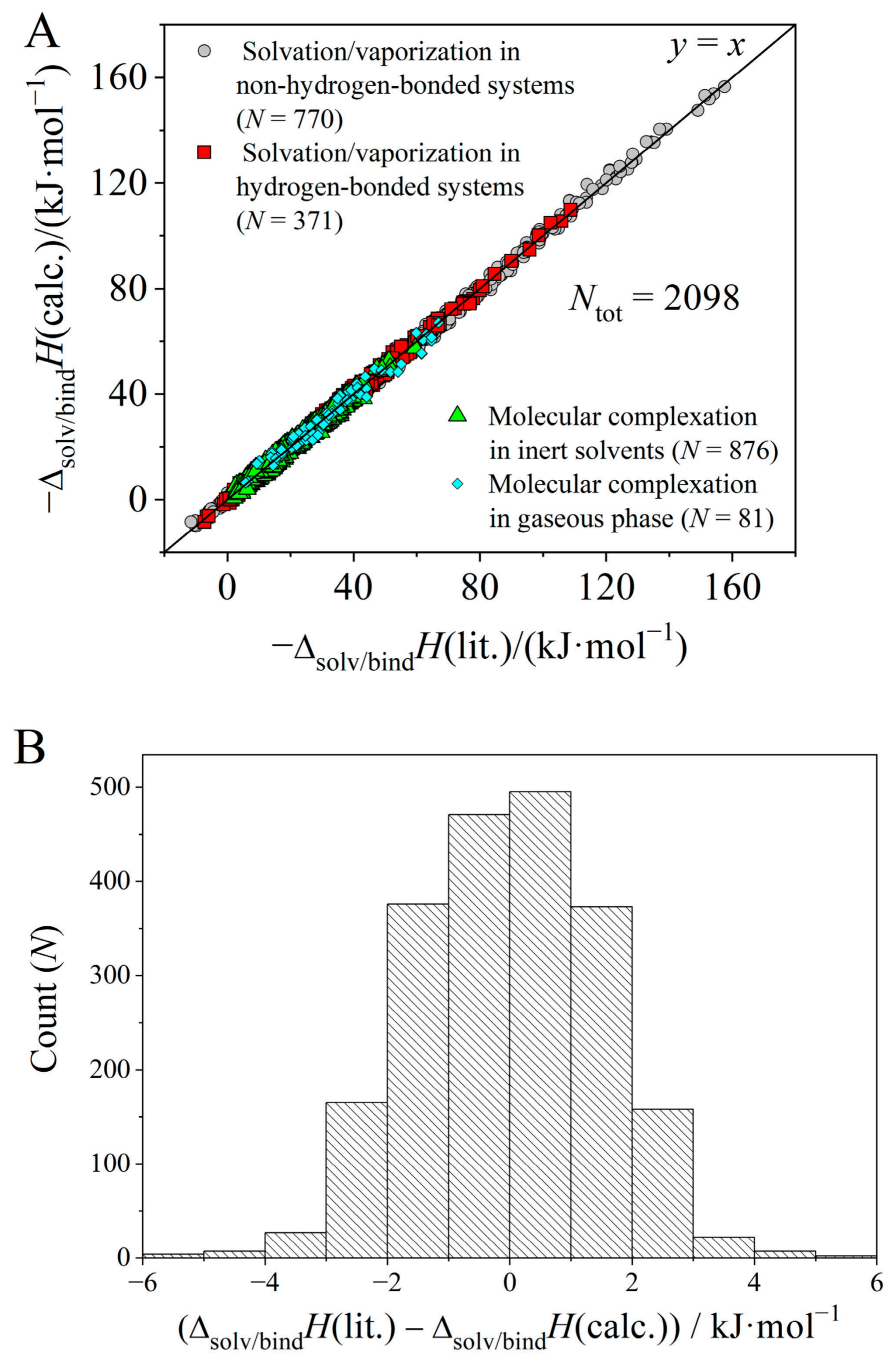
A comparison between the experimental solvation/binding enthalpies taken from the literature and the values calculated from the Gibbs energies at 298.15 K according to Equations (19), (22), (27), (29), (34), (39) and (45) (**A**). Histogram of the deviations between the literature and calculated enthalpy values (**B**).

In Figure 9A, the experimental enthalpy changes are compared with the values calculated from the Gibbs energies at 298.15 K for the solvation and molecular complexation processes discussed above. The deviation histogram is shown in Figure 9B.

One can see that, for 2098 diverse processes and systems considered, the Δ*H*(lit.) − Δ*H*(calc.) values are symmetric and bell-shaped, with *AD* and *rAD* of 0.0 kJ·mol^−1^ and 0.1%, respectively, and *RMSD* and *rRMSD* of 1.5 kJ·mol^−1^ and 4.6%. Figure 9B suggests that the deviations between the calculated and literature values are random, and those greater than 3 kJ·mol^−1^ might be related to the measurement features.

Still, we should highlight that sometimes the literature data are scattered beyond the experimental uncertainty and *RMSD* stated above. In such cases, simple averaging or weighting the available measurements would not be appropriate. When analyzing such data before [21,22], we attempted to unambiguously distinguish the deviations caused by measurement errors. Frequently, the experimental data significantly deviating from our predictions were also inconsistent with other studies available, as well as the measurements of structurally similar systems. In our analysis, we discarded such literature results, e.g., inconsistent data on HF dimerization and trifluoroacetic acid–acetone gas-phase binding [22]. In some cases, a justified, independent selection between the scattered data points was not possible. In Ref. [21], we compared the study of tetracyanoethylene–arenes complexation in dichloromethane solution [108] and its step-by-step reproduction [109]. The data from the more recent Ref. [109] were described by Equation (39) within 2 kJ·mol^−1^, while for Ref. [108], *RMSD* was 7.3 kJ·mol^−1^. In this case, the better agreement between the calculations and the novel work [109] was a reason to discard the preceding study [108].

The need for critical analysis and sometimes selection of the literature data, as well as inevitable experimental uncertainties, raises a question regarding the generality of the relationships between the Gibbs energies and enthalpies proposed above. What convinces us is their wide applicability, diversity of the systems, and absence of systematic patterns in the histogram in Figure 9B. Therefore, we believe that the revealed regularities have a number of fundamental outcomes.

First, enthalpy–entropy compensation, often observed in solutions, is often attributed to solvent reorganization effects [33,110,111,112,113,114]. Another hypothesized reason is group additivity in both enthalpy and entropy (or Gibbs energy) in a series of given processes [9,10], e.g., solvation. However, the common form of the relationships between Δ*G*, Δ*H*, and Δ*S* in solvation, vaporization, solution- and gas-phase complexation processes suggests the deeper roots for Equation (46). Still, it is clear that different Δ*G* vs. Δ*H* relations are possible, e.g., in bimolecular reactions [115], where the slope is one. Establishing the origin of the numerical constant 0.660, connecting the thermodynamics of various processes involving weak intermolecular interactions, is of future interest.

Second, over almost a century, different Δ*S* vs. Δ*H* (or Δ*G* vs. Δ*H*) relationships have been attributed to variations in interaction mechanisms [4]. Thus, compensation plots were used for a qualitative analysis of the thermodynamic functions within a narrow series of processes, from solvation and molecular complexation to extraction [116], adsorption and chromatographic retention [117,118,119,120], supra- and biomolecular recognition [13,15,121,122,123,124,125]. However, the findings presented above enable the use of the Gibbs equation in a novel way, extracting the two entropic contributions:

(46) $$\begin{array}{c} \Delta G=\Delta H-T\Delta S=H-T\Delta S(\mathrm{comp}.)-T\Delta S(\mathrm{noncomp}.)=\\ 0.660\cdot \Delta H-T\Delta S(\mathrm{noncomp}.) \end{array}$$

The first one depends on the interaction enthalpy (Δ*S*(comp.)), and the other defines the intercept of the Δ*G* − Δ*H* relation (Δ*S*(noncomp.)). We assume that the mechanism of interaction influences the Δ*S*(noncomp.) value and not the slope of the Δ*G* vs. Δ*H* relationship at 298.15 K, as long as the processes involving noncovalent bonding are analyzed. Although much less data are available for the analysis at temperatures other than 298.15 K, it is reasonable to expect that similar Δ*G* vs. Δ*H* relationships hold under different conditions, yet with varying slope and intercept. The Δ*G* vs. Δ*H* correlations for the vaporization process at varying temperatures can be obtained [126], considering that there is also a correlation between the heat capacity and enthalpy changes on vaporization [127,128,129]. The generalization and extension to more processes is a subject of future research.

We believe that Equation (46) can be fruitfully applied to many other phenomena involving weak interactions, which are ubiquitous in Chemistry and Biology [130]. Below we show how Equation (46) applies to a process far more mechanistically complex than considered above, namely, inclusion compound formation.

#### 3.3.2. The Relationships Between Δ*G*, Δ*H*, and Δ*S* of Supramolecular Recognition

Cyclodextrins are probably the most frequently mentioned as host molecules in supramolecular chemistry [131,132]. They are macrocyclic oligomers of *α-D*-glucose, resembling truncated cones with primary and secondary hydroxyl groups. Having a hydrophobic cavity, they can bind guests of appropriate size, forming inclusion complexes. It is believed [14,133] that inclusion complexation thermodynamics is contributed by: (1) electrostatic (Coulombic) interaction; (2) van der Waals interaction; (3) hydrophobic interaction; (4) hydrogen bonding; (5) conformational strain; (6) exclusion of the water molecules from the cavity; and (7) charge transfer.

In the previous sections, we essentially considered each of the above interaction types, except Coulombic interaction in electrolyte solutions. Thus, it is reasonable to expect that supramolecular complexation might be analyzed within the framework of the Barclay–Butler rule as well. Indeed, the relationships between the Gibbs energies, enthalpies, and entropies of supramolecular complexation have been studied for almost 50 years [134]. An important reason for such an interest is probably the reduced variation in Δ_bind_*G*^CD^ upon substantial change in an interaction energy due to enthalpy–entropy compensation, as mentioned in Section 1. The analysis of Δ_bind_*H*^CD^ vs. Δ_bind_*S*^CD^ relationships was performed in Refs. [14,135,136,137]. In a series of binding processes of a particular cyclodextrin at 298.15 K, Δ_bind_*H*^CD^ vs. Δ_bind_*S*^CD^ correlations with a slope (i.e., compensation temperature) of nearly 250–400 K have been obtained. Such a slope would correspond to a Δ_bind_*G*^CD^ vs. Δ_bind_*H*^CD^ correlation with a slope close to zero, meaning that there is almost complete enthalpy–entropy compensation. However, the latter may often be caused by measurement artifacts, as highlighted by Exner [138]. Indeed, one can encounter anomalously exothermic cyclodextrin complexes, yet moderately stable in terms of *K*_bind_, e.g., α-cyclodextrin adducts with polyarenes [139] and long-chained dicarboxylic acids [140]. The authors suggest the anomalies are caused by the change in stoichiometry, non-inclusional association, micellization, and other parallel processes, which may particularly misrepresent the measured thermodynamic parameters.

The possibility of adverse processes, together with other complications encountered in an experiment (Section 2.4), makes it difficult to analyze the relationships between Δ_bind_*G*^CD^, Δ_bind_*H*^CD^, and Δ_bind_*S*^CD^ of cyclodextrins. Nonetheless, we made such an attempt within the framework of the above results. Complexation processes of simple organic molecules in an inert solvent follow Equation (21) with a slope of 0.660 and an intercept of 2.5*n.* However, cyclodextrin complexation is typically studied in water, where hydrophobic interactions, conformational strain, and substitution of the water molecules from the cavity may contribute to the intercept. Prediction of the related effects would require in-depth knowledge of the host–guest interaction mechanism, which is not always known. Instead, we believe that the analysis of the deviations Δ_bind_*G*^CD^ − 0.660Δ_bind_*H*^CD^ might be, at first, a tool for studying macromolecular recognition and then a predictive instrument.

In this work, we analyzed the relationship between Δ_bind_*G*^CD^ and Δ_bind_*H*^CD^ of *α*-cyclodextrin, which is probably one of the most studied host molecules. In a comprehensive review by Rekharsky and Inoue [14], 488 pairs of Δ_bind_*G*^CD^ vs. Δ_bind_*H*^CD^ values at 298.15 K are available for *α*-cyclodextrin. The Δ_bind_*G*^CD^ (corrected to mole-fraction scale by subtracting 10.0 kJ·mol^−1^ from concentration-scale Δ_bind_*G*_c_^CD^, Equation (11)) values varied from −54.3 kJ·mol^−1^ to −9.0 kJ·mol^−1,^ and the Δ_bind_*H*^CD^ values varied from −80.3 to 2.0 kJ·mol^−1^. We built the histogram based on the Δ_bind_*G*^CD^ − 0.660Δ_bind_*H*^CD^ values (Figure S1). It has a bell-shaped form with a sharp maximum between −15.5 kJ·mol^−1^ and −5 kJ·mol^−1^, where 68% of the Δ_bind_*G*^CD^ − 0.660Δ_bind_*H*^CD^ data points (330 out of 488) are located. Thus, 68% of the data were within Δ_bind_*G*^CD^ − 0.660Δ_bind_*H*^CD^ of −10.2 ± 5.2 kJ·mol^−1^. The standard deviation of the (Δ_bind_*G*^CD^ − 0.660Δ_bind_*H*^CD^) differences is somewhat greater (8.1 kJ·mol^−1^), but it seems to be strongly contributed by the data corresponding to non-inclusion complexes or those with stoichiometry other than assumed during Δ_bind_*f*^CD^ calculation (1:1), as in Refs. [139,140]. At the same time, the average standard deviation of Δ_bind_*H*^CD^ values reproduced multiple times was ±5.2 kJ·mol^−1,^ and that of Δ_bind_*G*^CD^ was 1.9 kJ·mol^−1^ (Section 2.4), making the observed scatter in Δ_bind_*G*^CD^ − 0.660Δ_bind_*H*^CD^ magnitudes close to the combined measurement uncertainty.

What is especially surprising is that the Δ_bind_*G*^CD^ − 0.660Δ_bind_*H*^CD^ within the above range is met for strikingly diverse guests. In Table S7, we showed the data on 210 unique cyclodextrin–guest–medium systems, for which Δ_bind_*G*^CD^ − 0.660Δ_bind_*H*^CD^ values are between −13.6 kJ·mol^−1^ and −7.0 kJ·mol^−1^. This list was obtained by removing duplicate ligand–solvent systems unless a different medium (pH, electrolytes) is mentioned. The data were compiled from the review [14] and the papers [137,141,142,143,144,145,146,147,148,149,150,151,152,153,154] published afterwards. The representative examples, ranging from inorganic anions and non-polar organic non-electrolytes to bulky amphiphilic ions and biologically active molecules, are shown in Table 5.

The applicability of the linear Δ*G* vs. Δ*H* relationship with a slope of 0.660 to supramolecular recognition confirms that the thermodynamic regularities established for solvation and simple molecular complexation extend to macromolecular host–guest systems, enabling the prediction of binding enthalpies from single-temperature equilibrium data and offering a unified approach for interpreting intermolecular interactions in complex processes.

Although the wide range of experimentally studied processes covered by Equation (46) is impressive, one should keep in mind that there are exceptions. In particular, in hetero- and homolytic covalent bond dissociation processes [115], acid–base, electron-transfer reactions [155], the Gibbs energy and enthalpy changes exhibit correlations with a slope close to one. Less predictable relations can be observed in complex reactions, involving disruption and formation of multiple bonds and significant geometry changes. Among the processes involving the molecular compounds, the fusion [156] and sublimation [157] thermodynamic parameters at 298.15 K do not obey Equation (46) as well. We believe that the deviations from Equation (46) in these particular cases are fundamental for better understanding of the regularities in the Δ*G* vs. Δ*H* relationship in the near future.

## 4. Conclusions

The systematic analysis of more than 4500 experimental thermodynamic values demonstrates that the relationships between the Gibbs energy and enthalpy changes in solvation, vaporization, and molecular complexation processes are governed by predictable linear regularities. The diversity of the molecular architectures and intermolecular force types, the processes, and the phases they take place in suggest that their origin cannot be explained solely by artifacts and statistical effects or solvent reorganization. Apparently, it implies that there are fundamental restraints on the thermodynamics of intermolecular interactions that we still need to understand.

Thermodynamics of solvation in non-hydrogen-bonded systems is described by a single Δ*G* vs. Δ*H* dependence, first revealed by Barclay–Butler. Systematic deviations from this baseline are quantitatively explainable through distinct physical contributions: conformational flexibility in long-chain aliphatics modifies the entropic term due to hindered internal rotation in the condensed phase; specific hydrogen-bonding interactions introduce a consistent ~2.5*n* kJ·mol^−1^ entropic penalty per directional bond, arising from the statistical ordering of the solution mixture; and solvophobic effects in associated solvents operate almost exclusively through entropic cavity-formation contributions, with negligible enthalpic components except in water.

By transforming these solvation and vaporization rules within basic thermodynamic cycles, robust linear relationships with an invariant slope of 0.660 were derived for both solution-phase and gas-phase molecular complexation. These rules successfully describe hydrogen-bonding, charge transfer, and dispersion-driven interactions across an exceptionally wide range of binding strengths and molecular structures, validating their status as a general thermodynamic principle. Furthermore, applying this framework to α-cyclodextrin host–guest systems reveals that the majority of genuine inclusion complexes exhibit a characteristic deviation from the Barclay–Butler line: (Δ_bind_*G*^CD^ − 0.660Δ_bind_*H*^CD^) values were within (6.4–13) kJ·mol^−1^ for 210 diverse inclusion compounds. This clustering provides a practical diagnostic tool for identifying (1) interaction mechanisms and (2) anomalous data, non-inclusion binding modes, stoichiometric inconsistencies, or parallel processes in supramolecular recognition studies.

We conclude that there is indeed a general relationship between the Gibbs energy and enthalpy changes in the processes of solvation, vaporization, and complexation of molecular compounds. The invariant slope of 0.660 means that nearly 1/3 of the enthalpy change is compensated entropically. The rest of the entropy defines the intercept of the Δ*G* vs. Δ*H* relation. The discussed relationship enables the accurate prediction of binding and solvation enthalpies from single-temperature Gibbs energy measurements at 298.15 K, significantly reducing the experimental burden of multi-temperature van’t Hoff analyses, and offers a unified, quantitative framework for interpreting intermolecular interactions. These principles have promising extensions to complex chemical, biochemical, and materials science processes, providing a reliable foundation for molecular design, solvent optimization, and supramolecular engineering.

## Figures and Tables

**Table 1:** The Gibbs energies and enthalpies of vaporization (i.e., “desolvation” from itself) of perhydrochrysene, perhydrophenanthrene, and perhydroacenaphthene at 298.15 K.

Compound	Δ_vap_*G* (lit)	Δ_vap_*H* (lit)	Δ_vap_*H* (Equation (19))	Δ_vap_*H* (Equation (20))	Ref.
	kJ·mol^−1^				
perhydrochrysene	38.2	83.5	83.6	90.6	[54]
perhydrophenanthrene	27.6	67.1	67.6	71.3	[55]
perhydroacenaphthene	22.5	59.4	59.9	62.1	[55]

**Table 2:** The rotational contributions to the entropy due to rotation along the C-C bond at 298.15 K calculated using the Fourier grid Hamiltonian method.

I_red_, a.m.u. Å^2^	V(φ) from [62] (Butane C_2_-C_3_) ^a^			V(φ) from [61] (Averaged RCH_2_–CH_2_R) ^b^		
	S_rot_(g) ^a^	S_rot_(l) ^c^	Δ*S*_rot_	S_rot_(g) ^b^	S_rot_(l) ^c^	Δ*S*_rot_
	J·K^−1^·mol^−1^					
11	21.8	19.9	1.9	21.2	19.0	2.2
20	24.2	22.4	1.8	23.6	21.4	2.2
50	28.0	26.1	1.9	27.4	25.2	2.2
100	30.9	29.0	1.9	30.3	28.1	2.2
200	33.7	31.8	1.9	33.1	30.9	2.2
500	36.7	34.9	1.8	36.2	34.0	2.2
1000	37.8	35.9	1.9	37.2	35.0	2.2

^a^ *V*(*φ*) = 0.5Σ*V*_i_(1−cos(i*φ*)) with *V*_1_ = 242 cm^−1^, *V*_2_ = 43 cm^−1^, *V*_3_ = 1148 cm^−1^, *V*_4_ = 40 cm^−1^, *V*_5_ = −6 cm^−1^, *V*_6_ = −36 cm^−1^. Applied to the gas phase. ^b^ *V*(*φ*) = 0.5Σ*V*_i_(1−cos(i*φ*)) with *V*_1_ = 821 cm^−1^, *V*_2_ = −351 cm^−1^, *V*_3_ = 1151 cm^−1^, *V*_4_ = −46 cm^−1^, *V*_5_ = 29 cm^−1^, *V*_6_ = −34 cm^−1^. Applied to the gas phase. ^c^ *V*_i_(l) are assumed to be 1.25*V*_i_(g).

**Table 3:** The Gibbs energies and enthalpies of dimerization of carboxylic acids in benzene, lactams, and phenols in tetrachloromethane at 298.15 K.

Compound	−Δ_bind(S)_*G* (lit)	−Δ_bind(S)_*H* (lit)	−Δ_bind(S)_*H* (Equation (39)) ^a^	Δ ^b^	Ref.
	kJ·mol^−1^				
acetic acid	21.2	37.7	39.7 ^c^	2.0	[92]
chloroacetic acid	18.6	35.4	35.7 ^c^	0.3	[92]
bromoacetic acid	18.3	33.6	35.3 ^c^	1.7	[92]
iodoacetic acid	19.6	37.0	37.3 ^c^	0.3	[92]
propionic acid	21.7	41.5	40.5 ^c^	−1.0	[92]
2-chloropropionic acid	18.4	33.8	35.4 ^c^	1.6	[92]
3-chloropropionic acid	20.5	38.2	38.6 ^c^	0.4	[92]
*β*-lactam (2-azetidinone)	11.9	29.6	25.6 ^c^	−4.0	[93]
*γ*-lactam (2-pyrrolidone)	13.8	30.0	28.4 ^c^	−1.6	[93]
Δ-lactam (2-piperidinone)	14.1	28.3	28.9 ^c^	0.6	[93]
4-chlorophenol	7.8	13.4	15.5	2.1	[29]
4-methoxyphenol	8.5	15.9	16.7	0.8	[29]

^a^ The binding enthalpies calculated according to Equation (39) from the literature Gibbs energies of complex formation given in column 3. ^b^ The difference Δ_bind(S)_*H*(lit.) − Δ_bind(S)_*H* (Equation (39)). ^c^ *n* = 2 is considered in Equation (39) due to cyclic dimer formation in solution.

**Table 4:** The experimental Gibbs energies and enthalpies of gas-phase molecular complexation and the enthalpies calculated according to Equation (45).

Compound A	Compound B	Δ_bind_*G* (lit)	−Δ_bind_*H* (lit)	−Δ_bind_*H* (Equation (45)) ^a^	Δ ^b^	Ref.
		kJ·mol^−1^				
water	water	8.2	15.0	17.1	2.1	[96]
water	dimethyl ether	7.8	17.7	17.7	0	[97]
HF	HF	10.3	17.8	13.9	−3.9	[98]
HF	dimethyl ether	−6.5	43.0	39.4	−3.6	[99]
methanol	methanol	8.5	14.6	16.7	2.1	[100]
methanol	methylamine	1.6	23.4	27.1	3.7	[101]
methanol	dimethylamine	1.3	25.9	27.6	1.7	[101]
methanol	trimethylamine	0.4	28.9	28.9	0	[101]
methanol	triethylamine	−0.3	31.4	30.0	−1.4	[101]
1-propanol	1-propanol	5.0	22.0	22.0	0	[30]
2-butanol	2-butanol	4.6	22.0	22.6	0.6	[102]
trifluoroethanol	methanol	0.3	30.5	29.1	−1.4	[30]
acetic acid	acetic acid	−17.7	63.2	60.2	−3.0	[103]
formic acid	formic acid	−14.5	59.3	55.3	−4.0	[104]
		−14.6	63.8	55.5	−8.3	[103]
acetonitrile	acetonitrile	3.8	20.9	23.8	2.9	[30,105]

^a^ The binding enthalpies calculated according to Equation (45) from the literature Gibbs energies of complex formation given in column 3. ^b^ The difference Δ_bind(g)_*H*(lit) − Δ_bind(g)_*H* (Equation (45)).

**Table 5:** The comparison between the Gibbs energies and enthalpies of formation of representative inclusion compounds between *α*-cyclodextrin and molecular/ionic compounds at 298.15 K.

No.	Compound	−Δ_bind_*G*^CD^	−Δ_bind_*H*^CD^	−(Δ_bind_*G*^CD^ − −0.660 Δ_bind_*H*^CD^)	Medium	Method ^a^	Ref.
		kJ·mol^−1^					
1	acetic acid	15.5	11.2	8.1	H_2_O	pot	[14]
2	1-adamantylammonium (cation)	19.7	15.3	9.6	H_2_O (pH < 2.5)	uv	[14]
3	benzene	18.6	13.1	10.0	H_2_O	vap	[14]
4	6-bromo-2-naphthol	25.2	25.9	8.1	H_2_O	uv	[153]
5	cyclohexanone	17.7	11.1	10.4	H_2_O (pH 6.9)	cal	[14]
6	dimethyl terephthalate	25.0	22.0	10.5	H_2_O	fl	[147]
7	dodecyl trioxyethylenated sulfonate (anion)	30.9	32.8	9.3	H_2_O	cal	[151]
8	(*S*)-1-ferrocenylethanol	20.8	16.7	9.8	H_2_O (pH 11.4)	lc	[14]
9	1-heptanol	23.8	22.0	9.3	7M urea	cal	[141]
10	heptanoate (anion)	24.3	21.6	10.0	H_2_O	cal	[145]
11	heptanedioic acid	26.3	28.1	7.8	H_2_O (pH 1.3)	cal	[14]
12	4-[(4-hydroxy-3-propylphenyl)-azo]benzenesulfonate (dianion)	35.5	42.7	7.3	0.1M NaCl (pH 12)	uv	[14]
13	*L*-methionine (cation)	17.2	12.6	8.9	0.1 M H_2_SO_4_	uv	[137]
14	methyl orange (anion)	32.6	36.0	8.8	H_2_O (pH 7.5)	uv	[14]
15	4-nitrophenol	22.6	19.2	9.9	H_2_O (pH 3.0)	cal	[14]
16	3-octyl-1-methylimidazolium (cation)	27.5	24.0	11.7	H_2_O	uv	[154]
17	perchlorate (anion)	20.4	15.8	10.0	0.2 M NaCl	cal	[14]
18	triiodide (anion)	40.5	49.4	7.9	H_2_O (pH 11.0)	cal/uv	[14]
19	triethylamine	19.9	14.2	10.5	H_2_O	cal	[14]
20	undecanedioate	28.4	25.1	11.8	D_2_O (pD 13)	nmr	[14]

^a^ cal—calorimetry; fl—fluorimetry; lc—high-performance liquid chromatography; pot—potentiometry; uv—spectrophotometry; nmr—nuclear magnetic resonance.

## Data Availability

The data are contained within the article and Supplementary Materials.

## Supplementary Materials

The following supporting information can be downloaded at: https://www.mdpi.com/article/10.3390/molecules31193562/s1. Figure S1: Histogram showing the distribution of the compensation relationship (Δ_bind_*G*^CD^ − 0.660 Δ_bind_*H*^CD^) for inclusion compounds of α-cyclodextrins, demonstrating that most values fall between −13 kJ·mol^−1^ and −7 kJ·mol^−1^; Table S1: Comparison of vaporization enthalpies at 298.15 K calculated from Gibbs energies using Equation (19) with literature values for a wide range of non-associated organic non-electrolytes; Table S2: Comparison of solvation enthalpies at 298.15 K calculated from Gibbs energies using Equation (22) with literature values for solutes that form hydrogen bonds with non-associated solvents; Table S3: Coefficients (*k*_S_ and *b*_S_) for Equation (26), used to calculate the solvophobic contribution (Δ_s.e._G) for various associated solvents; Table S4: Comparison of solvation enthalpies at 298.15 K calculated from Gibbs energies using Equation (25) for organic non-electrolytes that are incapable of hydrogen bonding in associated solvents; Table S5: Comparison of solvation enthalpies at 298.15 K calculated from Gibbs energies using Equation (29) for organic non-electrolytes that form hydrogen bonds with associated solvents; Table S6: Comparison of solvation enthalpies at 298.15 K calculated from Gibbs energies for organic non-electrolytes that compete for hydrogen bonding with associated solvents; Table S7: Comprehensive list of molecular and ionic guests that form inclusion compounds with α-cyclodextrin, presenting their Gibbs energies and enthalpies of binding (Δ_bind_*G*^CD^ and Δ_bind_*H*^CD^), the value of their compensation relationship (Δ_bind_*G*^CD^ − 0.660 Δ_bind_*H*^CD^), the experimental medium, method, and references.
