# Supplementary material for: General Relationship Between Gibbs Energies and Enthalpies of Solvation, Vaporization, and Complexation for Molecular Compounds
Source: Molecules. 2026 Oct 7;31(19):3562. doi: 10.3390/molecules31193562 (PMC13667135; doi:10.3390/molecules31193562)
Supplement: Supplementary file 1 [file molecules-31-03562-s001.zip › molecules-4560182-supplementary.pdf]

Supporting Information

General Relationship between Gibbs Energies and Enthalpies of Solvation, Vaporization, and Complexation for Molecular Compounds

Table S1

The comparison of the vaporization enthalpies of non-associated organic non-electrolytes at 298.15 K calculated from the Gibbs energies according to Eq. (19) with the literature values.

No	compound	$\Delta_{vap}G$(lit)	$\Delta_{vap}H$(lit)	$\Delta_{vap}H$(Eq. 19)	Δ^{a}	Ref.
		kJ·mol^{-1}				
1	phenylphosphane	12.9±0.5	45.3±2.0	45.3	0.0	[1]
2	dichlorophenylphosphine	21.3±0.5	58.7±2.0	58.0	–0.7	[1]
3	4-fluorobenzaldehyde	16.4±0.5	52.2±2.0	50.6	–1.6	[1]
4	4-chloroanisole	18.3±0.5	54.5±2.0	53.4	–1.1	[1]
		18.4	54.1±2.0	53.7	–0.4	[2]
5	2-bromoanisole	21.7±0.5	60.9±2.0	58.6	–2.3	[1]
6	benzyl mercaptan	18.7±0.5	56.2±2.0	54.1	–2.1	[1]
7	4-methoxybenzenethiol	23.1±0.5	62.9±2.0	60.7	–2.2	[1]
8	1-chloro-4-(chloromethyl)benzene	21.1±0.5	59.2±2.0	57.7	–1.5	[1]
9	4-chloro(ethylbenzene)	16.8±0.5	52.7±2.0	51.2	–1.5	[1]
10	styrene oxide	18.3±0.5	55.6±2.0	53.5	–2.1	[1]
11	2′-chloroacetophenone	22.4±0.5	61.0±2.0	59.8	–1.2	[1]
12	4-ethylnitrobenzene	26.0±0.5	67.6±2.0	65.2	–2.4	[1]
13	1-phenylprop-1-yne	17.2±0.5	54.0±2.0	51.8	–2.2	[1]
14	4-ethynyltoluene	14.9±0.5	49.6±2.0	48.3	–1.3	[1]
15	4-methoxyphenylacetylene	20.7±0.5	59.3±2.0	57.1	–2.2	[1]
16	4-ethylanisole	17.9±0.5	54.0±2.0	52.9	–1.1	[1]
17	1,2-dimethoxy-4-methylbenzene	23.6±0.5	65.6±2.0	61.5	–4.1	[1]
18	dimethyl-p-toluidine	20.0±0.5	57.7±2.0	56.1	–1.6	[1]
19	4-isopropylbenzaldehyde	22.7±0.5	60.6±2.0	60.1	–0.5	[1]
20	mesitaldehyde	24.8±0.5	65.9±2.0	63.4	–2.5	[1]
21	1-bromo-4-tert-butylbenzene	22.4±0.5	60.6±2.0	59.7	–0.9	[1]
22	2-bromothiophene	12.5±0.5	45.4±2.0	44.7	–0.7	[1]
23	3-bromothiophene	13.3±0.5	46.6±2.0	45.9	–0.7	[1]
24	2-iodothiophene	16.9±0.5	53.4±2.0	51.3	–2.1	[1]
25	thiophene-2-carbonitrile	18.2±0.5	55.2±2.0	53.3	–1.9	[1]
26	thiophene-2-carbaldehyde	18.2±0.5	54.3±2.0	53.3	–1.0	[1]
27	2-acetylthiophene	21.1±0.5	59.5±2.0	57.7	–1.8	[1]

No	compound	$\Delta_{vap}G$(lit)	$\Delta_{vap}H$(lit)	$\Delta_{vap}H$(Eq. 19)	Δ[a]	Ref.
		kJ·mol^{-1}				
28	2-methylthiophene	8.3	37.8	38.4	0.6	[3]
29	3-methylthiophene	8.7	39.4	39.0	–0.4	[3]
30	2,5-di-methylthiophene	11.0	42.0	42.5	0.5	[3]
31	2,2′-di-thiophene	26.6	67.6	66.1	–1.5	[3]
32	2-methyl-1,3-benzoxazole	19.1±0.5	56.5±2.0	54.7	–1.8	[1]
33	2-methylbenzothiazole	23.8±0.5	63.1±2.0	61.9	–1.2	[1]
34	1-naphthaldehyde	31.1±0.5	73.8±2.0	72.9	–0.9	[1]
35	1-acetylnaphthalene	33.3±0.5	77.7±2.0	76.2	–1.5	[1]
36	4-phenoxytoluene	28.2±0.5	69.0±2.0	68.5	–0.5	[1]
37	*N*-methyldiphenylamine	29.5±0.5	69.9±2.0	70.4	0.5	[1]
38	benzyl ether	33.9±0.5	79.4±2.0	77.1	–2.3	[1]
39	2-chloroanisole	19.4	56.0±0.5	55.1	–0.9	[2]
40	3-chloroanisole	18.0	54.2±0.5	53.0	–1.2	[2]
41	2,3-dichloroanisole	24.8	64.9±0.5	63.3	–1.6	[2]
42	2,4-dichloroanisole	24.6	62.0±0.5	63.1	1.1	[2]
43	2,6-dichloroanisole	20.3	54.9±0.3	56.5	1.6	[2]
44	3,5-dichloroanisole	22.2	60.0±0.5	59.3	–0.7	[2]
45	4-iodoanisole	24.3	63.8±0.9	62.5	–1.3	[2]
46	methyl 2-methoxybenzoate	27.7±0.1	69.5±0.7	67.7	–1.8	[4]
47	methyl 4-methoxybenzoate	27.7±0.1	70.1±0.7	67.7	–2.4	[4]
48	*n*-ethylazide	2.1	28.8	29.0	0.2	[5] [b]
49	*n*-propyl azide	5.0	33.8	33.3	–0.5	[5] [b]
50	*n*-butyl azide	7.9	38.8	37.7	–1.1	[5] [b]
51	*n*-pentyl azide	10.7	43.8	42.0	–1.8	[5] [b]
52	*n*-hexyl azide	13.6	48.8	46.3	–2.5	[5] [b]
53	*n*-heptyl azide	16.4	53.8	50.6	–3.2	[5] [b]
54	*n*-octyl azide	19.3	58.8	55.6 [c]	–3.2	[5] [b]
55	*n*-nonyl azide	22.1	63.8	60.8 [c]	–3.0	[5] [b]
56	*n*-decyl azide	25.0	68.8	66.0 [c]	–2.8	[5] [b]
57	*n*-undecyl azide	27.9	73.8	71.2 [c]	–2.6	[5] [b]
58	*n*-dodecyl azide	30.7	78.8	76.4 [c]	–2.4	[5] [b]
59	*n*-tridecyl azide	33.6	83.8	81.6 [c]	–2.2	[5] [b]
60	*n*-tetradecyl azide	36.4±0.3	88.8	86.8 [c]	–2.0	[5] [b]
61	*n*-pentadecyl azide	39.3	93.8	92.0 [c]	–1.8	[5] [b]
62	*n*-hexadecyl azide	42.1	98.8	97.2 [c]	–1.6	[5] [b]
63	*n*-heptadecyl azide	45.0	103.8	102.4 [c]	–1.4	[5] [b]

No	compound	$\Delta_{vap}G$(lit)	$\Delta_{vap}H$(lit)	$\Delta_{vap}H$(Eq. 19)	Δ[a]	Ref.
		kJ·mol^{-1}				
64	*n*-octadecyl azide	47.8±0.3	108.8	107.5 [c]	–1.3	[5] [b]
65	*n*-nonadecyl azide	50.7	113.8	112.7 [c]	–1.1	[5] [b]
66	*n*-eicosyl azide	53.6	118.8	117.9 [c]	–0.9	[5] [b]
67	ethyl benzoate	20.7±0.3	60.7	57.7 [d]	–3.0	[6] [b]
68	propyl benzoate	23.2±0.3	64.9	62.0 [d]	–2.9	[6] [b]
69	butyl benzoate	25.8±0.3	69.2	66.4 [d]	–2.8	[6] [b]
70	pentyl benzoate	28.4±0.3	73.4	70.9 [d]	–2.5	[6] [b]
71	hexyl benzoate	31.1±0.3	77.7	75.4 [d]	–2.3	[6] [b]
72	heptyl benzoate	33.7±0.3	81.9	80.0 [d]	–1.9	[6] [b]
73	octyl benzoate	36.3±0.3	86.1	84.6 [d]	–1.5	[6] [b]
74	nonyl benzoate	39.0±0.3	90.4	89.1 [d]	–1.3	[6] [b]
75	decyl benzoate	41.6±0.3	94.6	93.8 [d]	–0.8	[6] [b]
76	undecyl benzoate	44.3±0.3	98.9	98.4 [d]	–0.5	[6] [b]
77	dodecyl benzoate	46.9±0.3	103.1	103.0 [d]	–0.1	[6] [b]
78	tridecyl benzoate	49.5±0.3	107.3	107.7 [d]	0.4	[6] [b]
79	tetradecyl benzoate	52.1±0.3	111.6	112.3 [d]	0.7	[6] [b]
80	pentadecyl benzoate	55.1±0.3	115.8	117.6 [d]	1.8	[6] [b]
81	hexadecyl benzoate	57.1±0.3	120.1	121.1 [d]	1.0	[6] [b]
82	heptadecyl benzoate	60.0±0.3	124.3	126.3 [d]	2.0	[6] [b]
83	octadecyl benzoate	62.6±0.3	128.5	131.0 [d]	2.5	[6] [b]
84	nonadecyl benzoate	65.3±0.3	132.8	135.7 [d]	2.9	[6] [b]
85	eicosyl benzoate	67.9±0.3	137.0	140.4 [d]	3.4	[6] [b]
86	2-decanone	21.2	61.0±0.8	59.7 [c]	–1.3	[7]
87	3-decanone	20.7	60.0±0.8	58.8 [c]	–1.2	[7]
88	4-decanone	20.3	60.7±0.4	58.0 [c]	–2.7	[7]
89	2-undecanone	24.4	65.6±0.8	65.5 [c]	–0.1	[7]
90	3-undecanone	23.3	65.2±0.5	63.4 [c]	–1.8	[7]
91	4-undecanone	23.3	65.7±0.6	63.4 [c]	–2.3	[7]
92	5-undecanone	23.1	65.6±0.5	63.2 [c]	–2.4	[7]
93	6-undecanone	23.4	65.2±0.6	63.7 [c]	–1.5	[7]
94	isophorone	19.6	56.2±0.4	56.8 [c]	0.6	[7]
95	*N,N*-dimethylformamide	13.1 [e]	46.4 [e]	45.6	–0.8	[8]
96	trimethyl phosphate	18.7	56.1	55.1 [c]	–1.0	[9]
97	triethyl phosphate	21.8	62.3	60.9 [c]	–1.4	[9]
98	dimethyl methylphosphonate	16.8	53.6	51.8 [c]	–1.8	[9]
99	diethyl methylphosphonate	18.5	56.9	54.8 [c]	–2.1	[9]

No	compound	$\Delta_{vap}G$(lit)	$\Delta_{vap}H$(lit)	$\Delta_{vap}H$(Eq. 19)	Δ[a]	Ref.
		kJ·mol^{-1}				
100	(-)-myrtenal	20.7	57.4	58.7	1.3	[10]
101	crimidine	32.7	75.6	75.4	–0.2	[11]
102	nitrapyrin	28.2	70.4	68.4	–2.0	[11]
103	4-chlorobenzaldehyde	20.3	56.7	56.6	–0.1	[12]
104	4-bromobenzaldehyde	22.9	61.2	60.5	–0.7	[12]
105	2,3-dichlorobenzaldehyde	24.2	62.7	62.4	–0.3	[12]
106	2,4-dichlorobenzaldehyde	23.4	61.9	61.2	–0.7	[12]
107	2,6-dichlorobenzaldehyde	26.0	66.2	65.1	–1.1	[12]
108	9-methylanthracene	39.4	83.2±2.3	85.5	2.3	[13]
109	phenazone	44.8	93.8	93.6	0.2	[14]
110	perhydrochrysene	38.2	83.5	83.6	–0.1	[15]
111	perhydrophenanthrene	27.6	67.1	67.6	–0.5	[16]
112	perhydroacenaphthene	22.5	59.4	59.9	–0.5	[16]

[a] The difference $\Delta_{vap}H$(Eq. (19) or (20)) – $\Delta_{vap}H$(lit.). [b] The literature data were obtained by the linear interpolation of the available measurements over homologous series. [c] Calculated according to Eq. (20). [d] Calculated by solving the equation given by the weighted sum of Eqs. (17) and (18). [e] Calculated using Cox equation provided by authors.

Calculation of the internal rotation contribution to the entropy of ideal gas and liquid phase butane

The internal rotation entropy was evaluated using the FGH 1D program [17]. The one-dimensional Schrödinger equation for the internal rotation was solved using a spatial grid of 250 points. The thermodynamic properties at 298.15 K were computed by including the lowest 250 energy levels in the partition function sum. Convergence of the calculated entropy was verified; expanding the grid size beyond 250 points or increasing the number of eigenstates included in the summation produced no meaningful change in the final entropy value.

Table S2

The comparison of the solvation enthalpies of organic non-electrolytes forming hydrogen bonds with non-associated solvents at 298.15 K calculated from the Gibbs energies according to Eq. (22) with the literature values. [18]

No	solute	solvent	$-\Delta_{solv}G$(lit)	$-\Delta_{solv}H$(lit)	$-\Delta_{solv}H$(Eq. 22)	Δ[a]
			kJ·mol^{-1}			
1	ethanol	dinonyl phthalate	3.7	34.3	35.2	0.9
2	methanol	dinonyl phthalate	3.5	33.7	34.8	1.1
3	trichloromethane	1-ethoxypropane	5.0	36.3	37.1	0.8
4	1-propanol	1-methyl-2-pyrrolidinone	10.4	45.2	45.3	0.1
5	ethanol	1-methyl-2-pyrrolidinone	7.7	42.5	41.2	–1.3
6	water	1-methyl-2-pyrrolidinone	11.4	51.1	50.5 [a]	–0.6
7	ethanol	isopropyl ether	1.6	30.4	32.0	1.6
8	trichloromethane	isopropyl ether	5.3	39.7	37.6	–2.1
9	1-propanol	tetraglyme	8.4	41.9	42.3	0.4
10	2-propanol	tetraglyme	6.3	40.5	39.1	–1.4
11	ethanol	tetraglyme	5.8	37.5	38.3	0.8
12	methanol	tetraglyme	4.7	35.0	36.6	1.6
13	trichloromethane	tetraglyme	8.4	42.9	42.2	–0.7
14	2-butanol	2-butanone	7.4	38.4	40.7	2.3
15	trichloromethane	2-butanone	5.8	36.5	38.3	1.8
16	2-propanol	*tert*-butyl ethyl ether	3.8	37.4	35.3	–2.1
17	methanol	2-octanone	1.7	34.0	32.1	–1.9
18	2-propanol	3-methyl-2-butanone	5.5	39.4	37.9	–1.5
19	trichloromethane	propylene carbonate	2.6	32.8	33.5	0.7
20	2-methyl-1-propanol	4-methyl-2-pentanone	9.0	44.5	43.1	–1.4
21	2-propanol	4-methyl-2-pentanone	5.5	40.8	37.9	–2.9
22	1-propanol	4-morpholine-carboxaldehyde	7.6	38.3	41.1	2.8
23	2-propanol	4-morpholine-carboxaldehyde	5.4	38.1	37.7	–0.4
24	2-methyl-1-propanol	isobutyl acetate	8.7	42.9	42.8	–0.1
25	acetic_acid	acetic anhydride	11.6	49.3	47.1	–2.2
26	1-propanol	butyl acetate	7.4	42.2	40.8	–1.4
27	methanol	butyl acetate	2.2	35.5	32.8	–2.7
28	water	butyl acetate	3.3	40.9	38.3 [a]	–2.6
29	trichloromethane	butyl acetate	6.1	38.8	38.8	0.0
30	methanol	propyl acetate	1.4	32.3	31.7	–0.6

31	water	propyl acetate	2.8	37.6	37.6 [a]	0.0
32	2-butanol	methyl butanoate	6.5	36.7	39.5	2.8
33	1-butanol	cyclohexanone	11.5	49.5	46.9	–2.6
34	1-pentanol	cyclohexanone	13.7	51.2	50.3	–0.9
35	water	dimethoxymethane	2.2	39.4	36.7 [a]	–2.7
36	methanol	*N,N*-dimethylacetamide	6.6	39.4	39.5	0.1
37	methanol	*N,N*-dimethylformamide	6.0	40.2	38.7	–1.5
38	trichloromethane	tri-*n*-pentylamine	5.1	38.3	37.2	–1.1
39	methanol	propyl propanoate	2.6	34.2	33.5	–0.7
40	methanol	tetrahydrofuran	2.4	35.6	33.2	–2.4

[a] The difference $\Delta_{solv}H$(lit.) – $\Delta_{solv}H$(Eq. 22). [b] In Eq. (22), $n = 2$ was taken.

Table S3

Coefficients of Eq. (26) used for calculating $\Delta_{s.e.}G$.

Solvent	k_S/(10^{-2} kJ cm^{-3})	b_S/(kJ mol^{-1})	Ref.
methanol	5.17	0.23	[19]
ethanol	3.98	0.8	[19]
1-propanol	3.94	0.36	[19]
1-butanol	3.0	0.50	[19]
1-pentanol	2.64	0.55	[20]
1-hexanol	1.82	0.5	[20]
1-heptanol	1.60	0.5	[20]
1-octanol	1.40	0.0	[21]
1-decanol	1.19	0.5	[20]
1-dodecanol	1.01	0.5	[20]
1-octadecanol	0.68	0.5	[20]
2-methylpropanol-1	2.50	0.5	[20]
3-methylbutanol-1	2.10	0.5	[20]
water	22.0	3.65	[19]
formamide	8.88	3.23	[22]
N-methylformamide	5.39	0	[23]
ethylene glycol	6.92	3.24	[24]
propylene glycol	4.91	1.5	[25]
2-methoxyethanol	2.87	0.12	[25]

Table S4. The comparison of the solvation enthalpies of organic non-electrolytes incapable of hydrogen bonding in associated solvents at 298.15 K calculated from the Gibbs energies according to Eq. (25) with the literature values.

No	solute	solvent	$-\Delta_{solv}G$ (lit)	$-\Delta_{solv}H$ (lit)	$\Delta_{s.e.}G^{a}$	$-\Delta_{solv}H$ (Eq. 27)	Δ^{b}	Ref.
			kJ·mol⁻¹					
1	hexane	*N*-methylformamide	–5.1	26.3	5.1	25.8	–0.5	[23]
2	heptane	*N*-methylformamide	–3.3	30.9	5.9	29.7	–1.2	[23]
3	octane	*N*-methylformamide	–1.1	35.1	6.7	34.2	–0.9	[23]
4	nonane	*N*-methylformamide	0.8	39.4	7.4	38.2	–1.2	[23]
5	decane	*N*-methylformamide	2.5	43.6	8.2	42.0	–1.6	[23]
6	cyclohexane	*N*-methylformamide	–2.7	28.5	4.6	28.6	0.1	[23]
7	*tert*-butylchloride	*N*-methylformamide	–2.3	27.5	4.3	28.8	1.3	[23]
8	benzene	*N*-methylformamide	1.2	32.7	3.9	33.5	0.8	[23]
9	toluene	*N*-methylformamide	3.3	36.4	4.6	37.7	1.3	[23]
10	ethylbenzene	*N*-methylformamide	5.1	40.4	5.4	41.7	1.3	[23]
11	*o*-xylene	*N*-methylformamide	6.1	40.6	5.4	43.2	2.6	[23]
12	*m*-xylene	*N*-methylformamide	5.3	40.3	5.4	42.0	1.7	[23]
13	*p*-xylene	*N*-methylformamide	5.2	40.1	5.4	41.8	1.7	[23]
14	fluorobenzene	*N*-methylformamide	2.2	34.7	4.0	35.2	0.5	[23]
15	chlorobenzene	*N*-methylformamide	6.1	39.9	4.5	41.8	1.9	[23]
16	naphthalene	*N*-methylformamide	14.4	53.7	5.9	56.5	2.8	[23]
17	neon	1-butanol	–22.1	–6.5	0.8	–6.5	0.0	[19]
18	argon	1-butanol	–17.4	2.4	1.1	1.1	–1.3	[19]
19	krypton	1-butanol	–14.9	4.9	1.3	5.2	0.3	[19]
20	xenon	1-butanol	–11.6	10.5	1.5	10.5	0.0	[19]
21	oxygen	1-butanol	–17.8	1.2	1.1	0.5	–0.7	[19]
22	methane	1-butanol	–15.6	3.8	1.3	4.1	0.3	[19]
23	ethane	1-butanol	–11.3	12.1	1.7	11.2	–0.9	[19]
24	propane	1-butanol	–8.2	17.7	2.1	16.5	–1.2	[19]
25	pentane	1-butanol	–2.6	25.7	3.0	26.4	0.7	[19]
26	hexane	1-butanol	0.0	30.2	3.4	30.9	0.7	[19]
27	heptane	1-butanol	2.6	34.8	3.8	35.5	0.7	[19]
28	octane	1-butanol	4.7	39.2	4.3	39.4	0.2	[19]
29	nonane	1-butanol	7.6	42.6	4.7	44.4	1.8	[19]
30	benzene	1-butanol	2.5	31.3	2.7	33.6	2.3	[19]
31	toluene	1-butanol	5.0	35.9	3.1	38.0	2.1	[19]
32	tetrafluoro-methane	1-butanol	–18.5	1.7	1.5	0.0	–1.7	[19]
33	tetrachloro-methane	1-butanol	2.3	32.6	2.8	33.5	0.9	[19]
34	*tert*-butyl chloride	1-butanol	–1.5	26.9	2.9	27.9	1.0	[19]

No	solute	solvent	$-\Delta_{solv}G$ (lit)	$-\Delta_{solv}H$ (lit)	$\Delta_{s.e.}G$[a]	$-\Delta_{solv}H$ (Eq. 27)	Δ[b]	Ref.
			kJ·mol^{-1}					
35	*tert*-butyl chloride	2-methoxyethanol	–0.4	26.8	2.4	28.8	2.0	[25]
36	benzene	2-methoxyethanol	3.0	33.2	2.2	33.6	0.4	[25]
37	bromobenzene	2-methoxyethanol	9.8	44.5	2.7	44.7	0.2	[25]
38	hexane	2-methoxyethanol	–2.9	24.6	2.9	25.8	1.2	[25]
39	heptane	2-methoxyethanol	–0.8	28.7	3.3	29.5	0.8	[25]
40	decane	2-methoxyethanol	5.5	40.6	4.5	40.9	0.3	[25]
41	biphenyl	2-methoxyethanol	22.2	64.2	3.9	65.3	1.1	[25]
42	methylcyclohexane	2-methoxyethanol	0.3	28.4	2.9	30.6	2.2	[25]
43	*m*-xylene	2-methoxyethanol	7.1	40.4	3.0	41.1	0.7	[25]
44	naphthalene	2-methoxyethanol	16.6	55.6	3.2	55.8	0.2	[25]
45	nonane	2-methoxyethanol	3.5	36.7	4.1	37.3	0.6	[25]
46	*o*-xylene	2-methoxyethanol	7.7	40.9	3.0	42.0	1.1	[25]
47	octane	2-methoxyethanol	1.6	32.8	3.7	33.8	1.0	[25]
48	*p*-xylene	2-methoxyethanol	6.9	40.3	3.0	40.8	0.5	[25]
49	toluene	2-methoxyethanol	4.9	36.8	2.6	37.1	0.3	[25]
50	undecane	2-methoxyethanol	7.3	44.6	4.9	44.2	–0.4	[25]
51	fluorobenzene	2-methoxyethanol	4.2	35.9	2.2	35.5	–0.4	[25]
52	chlorobenzene	2-methoxyethanol	7.6	41.3	2.5	41.1	–0.2	[25]
53	cyclohexane	2-methoxyethanol	–1.0	26.6	2.5	28.0	1.4	[25]
54	ethylbenzene	2-methoxyethanol	6.9	40.5	3.0	40.8	0.3	[25]

[a] Calculated according to Eq. (26) using the k_S and b_S values provided in Table S3. [b] The difference $\Delta_{solv}H$(lit.) – $\Delta_{solv}H$(Eq. 27).

Table S5. The comparison of the solvation enthalpies of organic non-electrolytes forming hydrogen bonds with associated solvents at 298.15 K calculated from the Gibbs energies according to Eq. (29) with the literature values.

No	solute	solvent	$-\Delta_{solv}G$ (lit)	$-\Delta_{solv}H$ (lit)	$\Delta_{s.e.}G^{a}$	$-\Delta_{solv}H$ (Eq. 29)	Δ^{b}	Ref.
			kJ·mol⁻¹					
1	1,2-ethanediol	1,2-propanediol	24.3	72.1	4.0	72.4	0.3	[18]
2	ethanol	1,2-propanediol	6.3	42.5	3.7	44.7	2.2	[18]
3	2-butanol	1-butanol	10.2	50.9	2.7	49.2	–1.7	[18]
4	2-methyl-2-butanol	1-butanol	10.2	49.6	3.2	49.8	0.2	[18]
5	2-propoxyethanol	1-butanol	16.4	59.3	3.3	59.5	0.2	[18]
6	ethanol	1-butanol	7.2	46.2	1.9	43.3	–2.9	[18]
7	methanol	1-butanol	4.6	37.4	1.4	38.7	1.3	[18]
8	1-decanol	1-dodecanol	27.8	75.4	2.1	74.9	–0.5	[18]
9	1-butanol	1-octadecanol	11.3	49.7	1.0	48.2	–1.5	[18]
10	1-propanol	1-octadecanol	8.7	44.6	0.9	44.1	–0.5	[18]
11	ethanol	1-octadecanol	6.5	43.0	0.8	40.7	–2.3	[18]
12	methanol	1-octadecanol	4.7	39.0	0.7	37.7	–1.3	[18]
13	ethanol	1-octanol	5.9	41.7	1.4	40.6	–1.1	[18]
14	methanol	1-octanol	3.9	36.9	1.1	37.2	0.3	[18]
15	ethanol	1-pentanol	6.2	41.8	1.7	41.5	–0.3	[18]
16	methanol	1-pentanol	4.2	39.0	1.4	38.0	–1.0	[18]
17	1-butanol	1-propanol	12.1	52.4	2.6	51.8	–0.6	[18]
18	1H-pyrrole	1-propanol	11.3	49.6	1.7	49.3	–0.3	[18]
19	2-methyl-1-butanol	1-propanol	13.8	56.0	3.0	55.0	–1.0	[18]
20	2-propen-1-ol	1-propanol	8.8	46.6	2.0	45.9	–0.7	[18]
21	2-propoxyethanol	1-propanol	16.2	59.1	2.2	57.4	–1.7	[18]
22	3-methyl-1-butanol	1-propanol	14.3	57.9	3.0	55.8	–2.1	[18]
23	methanol	1-propanol	5.0	41.2	1.3	39.0	–2.2	[18]
24	trichloromethane	1-propanol	3.1	35.8	2.2	37.7	1.9	[18]
25	1-propanol	ethanol	9.2	48.2	3.2	48.3	0.1	[18]
26	2-propanol	ethanol	7.6	47.2	3.2	45.9	–1.3	[18]
27	methanol	ethanol	4.8	38.9	2.1	39.9	1.0	[18]
28	1-butanol	ethanol	11.5	51.8	3.7	52.6	0.8	[26,27]
29	1-propanol	ethanol	9.0	46.2	3.2	48.0	1.8	[27,28]
30	2-propanol	ethanol	7.0	45.5	3.2	45.5	0.0	[27,28]

No	solute	solvent	$-\Delta_{solv}G$ (lit)	$-\Delta_{solv}H$ (lit)	$\Delta_{s.e.}G$[a]	$-\Delta_{solv}H$ (Eq. 29)	Δ[b]	Ref.
			kJ·mol⁻¹					
31	benzoic acid	ethanol	25.6	76.5	4.5	75.2	–1.3	[27,28]
32	1-pentanol	methanol	12.2	55.3	4.7	55.2	–0.1	[27,28]
33	benzoic acid	methanol	25.1	77.0	5.0	75.2	–1.8	[27,28]
34	ethanol	ethylene glycol	4.7	41.1	6.3	42.4	1.3	[27,28]
35	1-butanol	2-propanol	11.1	52.2	3.2	51.2	–1.0	[27,28]
36	1-decanol	2-propanol	26.3	80.0	6.6	79.4	–0.6	[27,28]
37	1-pentanol	2-propanol	14.0	56.8	3.8	56.5	–0.3	[27,28]
38	2-propanol	2-propanol	6.6	45.5	2.7	43.6	–1.9	[27,28]
39	methanol	2-propanol	3.6	37.5	1.6	37.4	–0.1	[27,28]
40	benzoic acid	2-propanol	25.8	74.8	4.0	74.7	–0.1	[27,28]
41	ethanol	2-propanol	5.6	42.2	2.1	41.2	–1.0	[27,28]
42	adipic acid	2-propanol	42.9	105.9	4.7	105.5	–0.4	[27,28]
43	1-pentanol	2-butanol	14.4	56.7	3.2	56.2	–0.5	[27,28]
44	1-propanol	2-butanol	8.0	46.3	2.3	45.2	–1.1	[27,28]
45	isobutanol	2-butanol	10.1	50.8	2.7	48.1	–2.7	[27,28]
46	2-butanol	2-butanol	9.1	50.1	2.7	47.4	–2.7	[27,28]
47	benzoic acid	2-butanol	26.1	74.7	3.3	74.1	–0.6	[27,28]
48	methanol	2-butanol	3.4	37.8	1.4	36.8	–1.0	[27,28]
49	ethanol	2-butanol	6.4	42.3	2.1	42.4	0.1	[27,28]
50	chloroform	2-butanol	3.0	35.5	2.4	37.7	2.2	[27,28]

[a] Calculated according to Eq. (26) using the ks and bs values provided in Table S3. [b] The difference $\Delta_{solv}H$(lit.) – $\Delta_{solv}H$(Eq. 29).

Table S6. The comparison of the solvation enthalpies of organic non-electrolytes competing for hydrogen bonding with associated solvents at 298.15 K calculated from the Gibbs energies according to Eq. (25) with the literature values.

No	Solute	Solvent	$-\Delta_{solv}G$ (lit)	$-\Delta_{solv}H$ (lit)	$\Delta_{s.e.}G$[a]	$-\Delta_{solv}H$ (Eq. (*))	Δ[b]	Ref.
1	pyridine	methanol	8.4	44.2	3.7	44.1	–0.1	[21]
2	2-methylpyridine	methanol	11.2	50.1	4.4	49.4	–0.7	[21]
3	3-methylpyridine	methanol	12.4	49.8	4.4	51.2	1.4	[21]
4	pyridine	ethanol	8.9	43.4	3.5	44.5	1.1	[21]
5	2-methylpyridine	ethanol	10.9	46.9	4.1	48.5	1.6	[21]
6	3-methylpyridine	ethanol	12.5	48.3	4.1	50.9	2.6	[21]
7	triethylamine	ethanol	5.4	44.0	5.0	41.5	–2.5	[21]
8	pyridine	1-butanol	8.7	40.4	2.6	42.9	2.5	[21]
9	2-methylpyridine	1-butanol	11.7	46.2	3.0	48.0	1.8	[21]
10	triethylamine	1-butanol	6.4	43.1	3.7	41.1	–2.0	[21]
11	butylamine	1-butanol	9.2	45.8	2.9	44.1	–1.7	[21]
12	pyridine	1-octanol	9.8	39.5	1.0	42.1	2.6	[21]
13	2-methylpyridine	1-octanol	13.2	45.5	1.2	47.6	2.1	[21]
14	1-butanol	formamide	5.9	48.5	9.5	49.1	0.6	[29,30]
15	1-hexanol	formamide	9.3	55.1	11.9	57.9	2.8	[29,30]
16	1-heptanol	formamide	10.8	60.2	13.1	62.0	1.8	[29,30]
17	1-pentanol	formamide	7.7	52.4	10.7	53.6	1.2	[29,30]
18	1-propanol	formamide	4.3	44.5	8.3	44.8	0.3	[29,30]
19	ethanol	formamide	4.3	40.1	7.1	43.0	2.9	[29,30]
20	methanol	formamide	2.9	36.6	5.9	39.1	2.5	[29,30]
21	water	formamide	7.8	42.8	4.7	44.7	1.9	[29,30]
22	2-pentanone	formamide	2.7	43.1	9.1	43.6	0.5	[29,30]
23	2-heptanone	formamide	5.2	51.5	11.5	51.1	–0.4	[29,30]
24	1,4-dioxane	formamide	4.1	40.6	6.6	42.0	1.4	[29,30]

[a] Calculated according to Eq. (26) using the k_S and b_S values provided in Table S3. [b] The difference $\Delta_{solv}H$(lit.) – $\Delta_{solv}H$(Eq. 29).

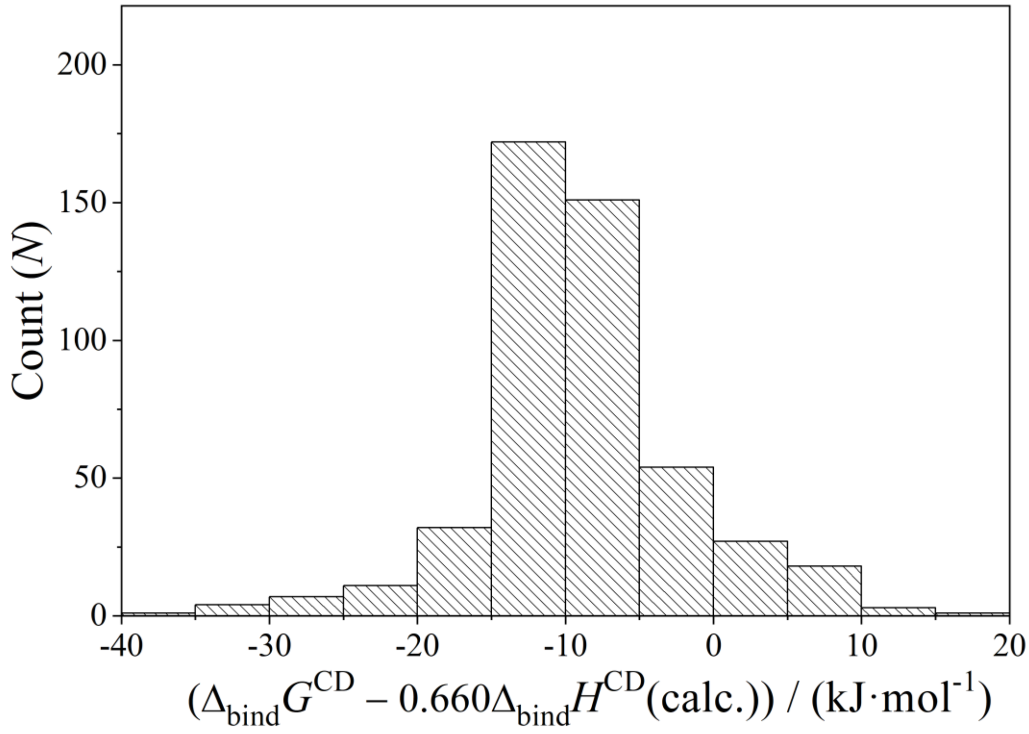

Fig. S1. Histogram of the $\Delta_{\mathrm{bind}}G^{\mathrm{CD}} - 0.660\ \Delta_{\mathrm{bind}}H^{\mathrm{CD}}$ values for inclusion compounds of α-cyclodextrins. The data on $\Delta_{\mathrm{bind}}G^{\mathrm{CD}}$ and $\Delta_{\mathrm{bind}}H^{\mathrm{CD}}$ values at 298.15 K are taken from Ref. [31].

Table S7. The comparison of the Gibbs energies and enthalpies of formation of inclusion compounds between α-cyclodextrin and molecular/ionic compounds at 298.15 K, demonstrating the diversity of guests for which $\Delta_{bind}G^{CD} - 0.660\ \Delta_{bind}H^{CD}$ is within 6–13 kJ·mol^{-1}.

No	compound	$-\Delta_{bind}G^{CD}$	$-\Delta_{bind}H^{CD}$	$-(\Delta_{bind}G^{CD} - 0.660\ \Delta_{bind}H^{CD})$	medium	method [a]	Ref.
		kJ·mol^{-1}					
1	acetic acid	15.5	11.2	8.1	H_2O	pot	[31]
2	acetonitrile	14.3	10.0	7.7	H_2O (pH 4.2)	pot	[31]
3	*N*-acetyl-*L*-leucinamide	17.4	6.1	13.4	H_2O	cal	[31]
4	1-adamantanecarboxylate (anion)	21.6	14.3	12.2	H_2O	cal	[31]
5	1-adamantylammonium (cation)	23.9	20.3	10.5	H_2O	pot	[31]
6	1-adamantylammonium (cation)	19.7	15.3	9.6	H_2O (pH < 2.5)	uv	[31]
7	adipate (dianion)	22.2	15.2	12.2	H_2O (pH 9.5)	cal	[31]
8	8-aminooctanoic acid	20.7	14.8	10.9	H_2O (pH 7.1)	cal	[31]
9	11-aminoundecanoic acid	29.0	26.6	11.4	H_2O (pH 6.7)	cal	[31]
10	*D*-amphetamine	17.9	12.6	9.6	H_2O (pH 11.0)	uv/cal	[31]
11	*L*-amphetamine	18.0	12.6	9.7	H_2O (pH 11.0)	uv/cal	[31]
12	8-anilino-1-naphthalene-sulfonate	18.0	10.5	11.1	H_2O (pH 2.0)	fl	[31]
13	benzene	18.6	13.1	10.0	H_2O	vap	[31]
14	1-bicyclo[2.2.1]heptane-carboxylate	17.5	13.0	8.9	H_2O (pH 8.5)	cal	[31]
15	1-bicyclo[2.2.1]hept-2-enecarboxylate	18.0	10.0	11.4	H_2O (pH 8.5)	cal	[31]
16	1-bicyclo[2.2.2]octane-carboxylate	18.4	14.2	9.0	H_2O (pH 8.5)	cal	[31]
17	bromodiphenhydramine hydrochloride	27.9	27.3	9.9	H_2O	cal	[31]
18	6-bromo-2-naphthol	25.2	25.9	8.1	H_2O	uv	[32]
19	4-bromophenol	26.3	25.6	9.4	H_2O (pH 4.2)	cal	[31]
20	1,2-butanediol	16.3	7.7	11.2	H_2O	cal	[31]
21	1,3-butanediol	15.7	6.7	11.3	H_2O	cal	[31]
22	1,4-butanediol	16.1	8.0	10.8	H_2O	cal	[31]
23	butanoate anion	16.3	10.6	9.3	H_2O (pH 6.9)	cal	[31]
24	butanoic acid	22.0	22.0	7.5	H_2O	cal	[33]
25	(*R*)-(-)-2-butanol	17.9	11.0	10.6	H_2O (pH 6.9)	cal	[31]
26	(*S*)-(+)-2-butanol	17.9	11.1	10.6	H_2O (pH 6.9)	cal	[31]
27	(±)-2-butanol	18.2	9.9	11.7	H_2O (pH 6.9)	cal	[31]
28	1-butanol	20.9	11.6	13.2	H_2O (pH 6.9)	cal	[31]
29	2-butanol	18.3	9.0	12.4	H_2O	cal	[31]
30	2-butanone	15.9	11.0	8.6	0.05 M HCl	uv	[31]
31	butylamine	21.6	15.1	11.6	H_2O	cal	[31]

No	compound	$-\Delta_{bind}G^{CD}$	$-\Delta_{bind}H^{CD}$	$-(\Delta_{bind}G^{CD} - 0.660\ \Delta_{bind}H^{CD})$	medium	method [a]	Ref.
		kJ·mol-1					
32	butylammonium (cation)	16.6	9.1	10.6	H_2O (pH 6.9)	cal	[31]
33	1-butylimidazole	22.8	20.4	9.3	H_2O (pH 10.0)	cal	[31]
34	chlorcyclizine	27.6	30.1	7.7	H_2O	pot	[31]
35	chlorcyclizine·2HCl	27.4	23.4	12.0	H_2O	cal	[31]
36	chlorcyclizine·HCl	27.4	24.4	11.3	H_2O	cal	[31]
37	2-chloro-4-[(4-hydroxy-phenyl)azo]benzoate	33.7	37.0	9.3	H_2O	cal	[31]
38	4-chlorophenol	24.1	20.1	10.8	H_2O (pH 4.2)	cal	[31]
39	3-cyanophenol	21.6	13.0	13.0	H_2O (pH 8.5)	uv	[34]
40	3-cyanophenolate (anion)	25.7	27.0	7.9	H_2O (pH 8.5)	uv	[34]
41	4-cyanophenol	21.3	19.2	8.6	H_2O (pH 4.0)	cal	[31]
41	4-cyanophenol	21.5	21.0	7.6	H_2O (pH 8.5)	uv	[34]
43	4-cyanophenolate (anion)	26.1	20.0	12.9	H_2O (pH 8.5)	uv	[34]
44	4-cyanophenolate	25.9	25.1	9.3	H_2O (pH 10.0)	cal	[31]
45	cyclobutanol	18.5	11.5	10.9	H_2O (pH 6.9)	cal	[31]
46	cycloheptanol	20.5	12.5	12.3	H_2O (pH 6.9)	cal	[31]
47	*cis*-1,2-cyclohexanediol	16.9	11.0	9.6	H_2O (pH 6.9)	cal	[31]
48	*trans*-1,2-cyclohexanediol	17.0	11.0	9.7	H_2O (pH 6.9)	cal	[31]
49	cyclohexanol	20.3	14.0	11.1	H_2O	uv	[31]
50	cyclohexanol	20.2	13.0	11.6	H_2O (pH 4.2)	pot	[31]
51	cyclohexanol	20.1	13.3	11.3	H_2O (pH 6.9)	cal	[31]
52	cyclohexanone	17.7	11.1	10.4	H_2O (pH 6.9)	cal	[31]
53	cyclopentanol	18.9	11.5	11.3	H_2O (pH 6.9)	cal	[31]
54	1,10-decanediol	32.0	31.0	11.5	0.1 M H_2SO_4 + 0.5 M Na_2SO_4	uv	[31]
55	diethylamine	18.8	12.6	10.5	H_2O	cal	[31]
56	dimethyl terephthalate	25.0	22.0	10.5	H_2O	fl	[35]
57	dimethyl 2,3-naphthalenedicarboxylate	17.7	9.1	11.7	H_2O	fl	[36]
58	dihexyl 2,6-naphthalenedicarboxylate	22.7	23.6	7.1	H_2O	fl	[37]
59	(2,5-dimethoxyphenethyl)-ammonium	18.2	13.6	9.2	H_2O (pH 5.0)	cal	[31]
60	(3,4-dimethoxyphenethyl)-ammonium	15.1	9.0	9.2	H_2O (pH 6.9)	cal	[31]
61	2-[[4-(dimethylamino)-phenyl]azo]pyridine	29.4	29.0	10.3	H_2O (pH 6.5-7.5)	kin	[31]
62	1,4-dioxane	13.7	10.0	7.1	H_2O (pH 4.2)	pot	[31]
63	diphenhydramine·HCl	19.4	16.2	8.7	H_2O	cal	[31]
64	dodecyl trioxyethylenated sulfonate (anion)	30.9	32.8	9.3	H_2O	cal	[38]
65	(1*R*,2*S*)-(-)-ephedrine	16.1	12.0	8.2	H_2O (pH 5.0)	cal	[31]
66	(1*S*,2*R*)-(+)-ephedrine	17.2	15.0	7.3	H_2O (pH 6.9)	cal	[31]
67	ethanol	13.5	3.0	11.5	H_2O (pH 4.2)	pot	[31]

No	compound	$-\Delta_{bind}G^{CD}$	$-\Delta_{bind}H^{CD}$	$-(\Delta_{bind}G^{CD} - 0.660\ \Delta_{bind}H^{CD})$	medium	method [a]	Ref.
		kJ·mol-1					
68	(ferrocenylmethyl) trimethylammonium (cation)	23.2	15.9	12.7	0.05 M NaCl	cal	[31]
69	(*R*)-1-ferrocenylethanol	21.5	20.9	7.7	H_2O (pH 11.4)	lc	[31]
70	(*S*)-1-ferrocenylethanol	20.8	16.7	9.8	H_2O (pH 11.4)	lc	[31]
71	4-fluorophenol	18.9	15.8	8.5	H_2O (pH 4.0)	uv	[31]
72	formic acid	13.5	3.1	11.5	H_2O	pot	[31]
73	heptanedioate (dianion)	17.9	10.1	11.2	H_2O (pH 11.3)	cal	[31]
74	heptanedioic acid	26.3	28.1	7.8	H_2O (pH 1.3)	cal	[31]
75	1,7-heptanediol	24.3	22.1	9.7	H_2O	cal	[31]
76	(*R*)-(-)-2-heptanol	26.4	22.1	11.8	H_2O (pH 6.90)	cal	[31]
77	(*S*)-(+)-2-heptanol	26.4	22.1	11.8	H_2O (pH 6.90)	cal	[31]
78	1-heptanol	24.7	20.6	11.1	H_2O	cal	[39]
79	1-heptanol	23.8	22.0	9.3	7M urea	cal	[39]
80	heptanoate (anion)	24.3	21.6	10.0	H_2O	cal	[33]
81	1-heptylamine	26.6	19.7	13.6	H_2O	cal	[40]
82	1-heptylammonium (cation)	25.6	19.9	12.5	H_2O	cal	[40]
83	1-heptylammonium (cation)	25.1	18.7	12.8	H_2O (pH 6.9)	cal	[31]
84	hydrogen *trans,trans*-2,4-hexadienedioate (anion)	20.4	18.0	8.5	H_2O	pot	[31]
85	1,5-hexanediol	18.8	13.0	10.2	H_2O	cal	[31]
86	1,6-hexanediol	21.3	16.1	10.7	H_2O	cal	[31]
87	(*R*)-2-hexanol	24.5	17.2	13.1	H_2O (pH 6.90)	cal	[31]
88	(*S*)-2-hexanol	24.5	16.9	13.3	H_2O (pH 6.90)	cal	[31]
89	1-hexanol	24.1	22.7	9.1	H_2O	cal	[41]
90	2-hexanol	22.7	15.0	12.8	H_2O	cal	[31]
91	3-hexanol	22.5	13.8	13.4	H_2O	cal	[31]
92	*trans*-2-hexenoate	24.0	17.2	12.6	H_2O (pH 6.9)	cal	[31]
93	*trans*-3-hexenoate	22.6	14.1	13.3	H_2O (pH 6.9)	cal	[31]
94	hexylammonium (cation)	24.7	17.6	13.1	H_2O (pH 6.9)	cal	[31]
95	3-hexyl-1-methylimidazolium cation	26.1	20.1	12.8	H_2O	uv	[42]
96	hydroquinone	17.9	10.0	11.3	H_2O (pH 4.2)	cal	[31]
97	4-[(4-hydroxy-3-chlorophenyl)-	25.1	27.0	7.3	H_2O (pH 11.1)	kin	[31]
98	trans-4-hydroxycinnamic acid	28.8	30.1	8.9	H_2O (pH 1.6)	uv	[31]
99	4-[(4-hydroxy-3,5-diisopropyl-phenyl)azo]benzenesulfonate	26.4	19.6	13.5	H_2O (pH 4.6)	kin	[31]
100	4-[(4-hydroxy-3,5-diisopropyl-phenyl)azo]benzenesulfonate (dianion)	26.6	21.9	12.1	H_2O	kin	[31]
101	5-[(4-hydroxy-3-ethylphenyl)-azo]naphthalene-1-sulfonate	23.1	18.8	10.7	H_2O (pH 11.1)	kin	[31]

No	compound	$-\Delta_{bind}G^{CD}$	$-\Delta_{bind}H^{CD}$	$-(\Delta_{bind}G^{CD} - 0.660\ \Delta_{bind}H^{CD})$	medium	method [a]	Ref.
		kJ·mol^{-1}					
102	hexyltrimethylammonium (cation)	23.9	16.1	13.3	H_2O	cal	[31]
103	3-[(4-hydroxyphenyl)azo]benzoate	31.2	33.4	9.2	H_2O	cal	[31]
104	4-[(4-hydroxyphenyl)azo]benzoate	30.7	29.1	11.5	H_2O	cal	[31]
105	4-[(4-hydroxy-3-propylphenyl)-azo]benzenesulfonate	32.6	33.3	10.6	H_2O (pH 4.6)	kin	[31]
106	4-[(4-hydroxy-3-propylphenyl)-azo]benzenesulfonate (dianion)	35.5	42.7	7.3	0.1M NaCl (pH 12)	uv	[31]
107	4-[(4-hydroxy-3-propylphenyl)-azo]benzenesulfonate (dianion)	32.3	31.8	11.3	H_2O	kin	[31]
108	5-[(4-hydroxy-3-propylphenyl)-azo]naphthalene-1-sulfonate	23.5	20.4	10.0	H_2O (pH 4.6)	kin	[31]
109	5-[(4-hydroxy-3-propylphenyl)-azo]naphthalene-1-sulfonate (dianion)	24.4	17.9	12.6	H_2O (pH 11.1)	kin	[31]
110	hydroxyzine·2HCl	27.5	21.1	13.6	H_2O	cal	[31]
111	hydroxyzine·HCl	27.5	22.0	13.0	H_2O	cal	[31]
112	4-iodoaniline	29.7	34.3	7.1	H_2O	pot	[31]
113	4-iodoanilinium	27.3	26.4	9.9	H_2O	pot	[31]
114	4-iodophenol	26.8	26.3	9.4	H_2O	cal	[31]
115	*L*-mandelic acid	23.0	21.0	9.1	H_2O	cal	[31]
116	*D*-mannose	9.0	0.0	9.0	H_2O	cal	[31]
117	meclizine·2HCl	26.8	22.7	11.8	H_2O	cal	[31]
118	methapyriline·HCl	20.6	16.0	10.0	H_2O (pH 7.0)	cal	[31]
119	*L*-methionine (cation)	17.2	12.6	8.9	0.1 M H_2SO_4	uv	[43]
120	(3-methoxyphenethyl) ammonium	17.0	14.6	7.4	H_2O (pH 5.0)	cal	[31]
121	(4-methoxyphenethyl) ammonium	18.4	15.0	8.5	H_2O (pH 5.0)	cal	[31]
122	4-methoxyphenol	20.0	19.5	7.1	H_2O (pH 4.0)	uv	[31]
123	methyl orange (acid form)	26.7	25.0	10.2	0.1 M H_2SO_4 +0.5 M Na_2SO_4	uv	[31]
124	methyl orange (anion)	32.6	36.0	8.8	H_2O (pH 7.5)	uv	[31]
125	methyl red (cation)	30.6	27.3	12.6	H_2O (pH 1.0)	uv	[31]
126	(+)-α-methylbenzylamine	18.1	15.1	8.1	H_2O (pH 11.0)	uv/cal	[31]
127	(-)-α-methylbenzylamine	18.7	15.5	8.5	H_2O (pH 11.0)	uv/cal	[31]
128	(±)-2-methylbutanoate	17.8	12.5	9.6	H_2O (pH 6.9)	cal	[31]

No	compound	$-\Delta_{bind}G^{CD}$	$-\Delta_{bind}H^{CD}$	$-(\Delta_{bind}G^{CD} - 0.660\ \Delta_{bind}H^{CD})$	medium	method [a]	Ref.
		kJ·mol^{-1}					
129	(1-methylbutyl)ammonium	17.4	11.2	10.0	H_2O (pH 6.9)	cal	[31]
130	*N*-methylbutylammonium	17.0	10.7	9.9	H_2O (pH 6.9)	cal	[31]
131	*trans*-3-methylcinnamate (anion)	22.1	21.8	7.7	H_2O (pH 8.2)	uv	[31]
132	*trans*-2-methylcinnamic acid	23.9	21.8	9.5	H_2O (pH 1.6)	uv	[31]
133	*trans*-3-methylcinnamic acid	29.9	29.7	10.3	H_2O (pH 1.6)	uv	[31]
134	2-methylcyclohexanone	18.4	8.9	12.5	H_2O (pH 6.9)	cal	[31]
135	1-methylnaphthoate	18.7	15.0	8.8	H_2O	fl	[44]
136	4-methoxytyramine	14.1	10.0	7.5	H_2O (pH 6.9)	cal	[31]
137	(1-methylhexyl)ammonium (cation)	25.1	17.9	13.3	H_2O (pH 6.9)	cal	[31]
138	*N*-methylhexylammonium (cation)	24.7	17.6	13.1	H_2O (pH 6.9)	cal	[31]
139	(4-methylphenethyl) ammonium (cation)	20.2	16.5	9.3	H_2O (pH 6.9)	cal	[31]
140	(4-ethylphenethyl)ammonium (cation)	19.4	13.9	10.2	H_2O (pH 5.0)	cal	[31]
141	*N*-methylphenethylammonium (cation)	16.5	11.8	8.7	H_2O (pH 5.0)	cal	[31]
142	4-methylphenol	20.0	16.8	8.9	H_2O (pH 4.0)	uv	[31]
143	3-methylphenyl acetate	20.1	18.0	8.2	H_2O (pH 10)	kin	[31]
144	2-methyl-1-propanol	17.7	9.4	11.5	H_2O	cal	[31]
145	2-methyl-2-propanol	18.4	12.0	10.5	H_2O	uv	[31]
146	monobutylurea	22.0	13.0	13.4	H_2O	cal	[31]
147	monoethylurea	12.6	7.4	7.7	H_2O	cal	[31]
148	monopropylurea	16.3	11.2	8.9	H_2O	cal	[31]
149	4-nitrophenol	22.6	19.2	9.9	H_2O (pH 3.0)	cal	[31]
150	4-nitrophenolate (anion)	22.2	20.2	8.9	1 M NaOH	cal	[31]
151	3-nitrophenyl acetate	19.8	16.0	9.2	H_2O (pH 10.6)	kin	[31]
152	nonanedioate	26.2	22.6	11.3	D_2O (pD 13)	nmr	[31]
153	1,9-nonanediol	29.8	27.0	12.0	0.1 M H_2SO_4 +0.5 M Na_2SO_4	uv	[31]
154	1,9-nonanediol	26.7	29.0	7.6	H_2O	cal	[45]
155	1,9-nonanediol	28.3	32.2	7.0	3M glucose	cal	[45]
156	nonanoate (anion)	27.0	26.8	9.3	H_2O	cal	[33]
157	2-norbornaneacetate	21.3	14.2	11.9	H_2O (pH 8.5)	cal	[31]
158	*D*-norleucine	19.5	9.3	13.4	H_2O	cal	[31]
159	octanedioate (dianion)	22.6	16.7	11.6	H_2O	pot	[31]
160	octanedioate (dianion)	21.2	11.8	13.4	H_2O (pH 11.3)	cal	[31]
161	1,8-octanediol	27.6	22.4	12.8	H_2O	cal	[31]
162	1,8-octanediol	27.8	25.0	11.3	0.1 M H_2SO_4 +0.5 M Na_2SO_4	uv	[31]

No	compound	$-\Delta_{bind}G^{CD}$	$-\Delta_{bind}H^{CD}$	$-(\Delta_{bind}G^{CD} - 0.660\ \Delta_{bind}H^{CD})$	medium	method [a]	Ref.
		kJ·mol^{-1}					
163	1,8-octanediol	27.8	25.0	11.3	3M glucose	cal	[45]
164	octanoate (anion)	26.2	26.4	8.8	H_2O	cal	[33]
165	1-octanol	25.0	22.0	10.5	H_2O	cal	[39]
166	3-octyl-1-methylimidazolium cation	27.5	24.0	11.7	H_2O	uv	[42]
167	hydrogen oxalate (anion)	12.2	-1.0	12.9	H_2O	pot	[31]
168	tris(pentafluoroethyl)-trifluorophosphate (anion)	24.3	23.4	8.9	H_2O	uv	[42]
169	pentakis(ethyleneglycol) monohexyl ether	22.9	16.9	11.7	H_2O	cal	[31]
170	pentanedioic acid	23.8	25.4	7.0	H_2O (pH 1.3)	cal	[31]
171	1,2-pentanediol	20.8	11.5	13.2	H_2O	cal	[31]
172	1,5-pentanediol	18.5	14.3	9.1	H_2O	cal	[31]
173	pentanoate	20.9	11.5	13.3	H_2O (pH 6.9)	cal	[31]
174	(*R*)-(-)-2-pentanol	22.0	15.4	11.8	H_2O (pH 6.9)	cal	[31]
175	(*S*)-(+)-2-pentanol	22.0	15.2	12.0	H_2O (pH 6.9)	cal	[31]
176	2-pentanol	21.4	13.4	12.6	H_2O	cal	[31]
177	3-pentanol	20.5	12.8	12.1	H_2O	cal	[31]
178	pentylammonium (cation)	21.2	13.6	12.2	H_2O (pH 6.9)	cal	[31]
179	perchlorate (anion)	20.4	15.8	10.0	0.2 M NaCl	cal	[31]
180	phenethylammonium (cation)	16.4	13.3	7.6	H_2O (pH 6.9)	cal	[31]
181	phenethylammonium (cation)	16.0	13.2	7.3	H_2O (pH 5.0)	cal	[31]
182	phenol	18.9	13.0	10.3	H_2O (pH 4.2)	pot	[31]
183	*L*-phenylalanine (anion)	18.0	13.0	9.4	H_2O (pH 11.3)	cal	[31]
184	*L*-phenylalanine	16.5	7.7	11.4	H_2O	cal	[31]
185	3-phenylpropionate	18.5	15.5	8.3	H_2O (pH 6.9)	cal	[31]
186	*L*-phenyltrifluoroethanol	19.9	12.6	11.6	H_2O (pH 11.0)	uv/cal	[31]
187	*D*-phenyltrifluoroethanol	19.5	14.6	9.9	H_2O (pH 11.0)	uv/cal	[31]
188	(3-phenylpropyl)ammonium (cation)	17.6	15.9	7.1	H_2O (pH 5.0)	cal	[31]
189	piroxicam	19.6	15.7	9.2	H_2O (pH 7.0)	cal	[31]
190	proadifen·HCl	22.9	20.8	9.2	H_2O	cal	[31]
191	1,2-propanediol	12.7	2.0	11.4	H_2O	cal	[31]
192	1,3-propanediol	13.6	6.7	9.2	H_2O	cal	[31]
193	1-propanol	17.9	11.0	10.6	0.05 M HCl	uv	[31]
194	2-propanol	13.8	2.0	12.5	H_2O (pH 4.2)	pot	[31]
195	propylamine	19.5	14.6	9.9	H_2O	cal	[31]
196	(1*S*,2*S*)-(+)-pseudoephedrine	16.7	13.7	7.7	H_2O (pH 5.0)	cal	[31]
197	pyrene	22.4	13.4	13.6	H_2O	lc	[31]
198	pyrilammonium maleate	21.8	19.1	9.2	H_2O (pH 7.0)	cal	[31]
199	resorcinol	16.9	10.7	9.8	H_2O	cal	[31]
200	*trans*-4,4'-bis(dimethylammoniomethyl) stilbene	28.0	26.8	10.3	H_2O	cal	[31]

No	compound	$-\Delta_{bind}G^{CD}$	$-\Delta_{bind}H^{CD}$	$-(\Delta_{bind}G^{CD} - 0.660\ \Delta_{bind}H^{CD})$	medium	method [a]	Ref.
		kJ·mol^{-1}					
201	thenyldiamine·HCl	21.5	13.9	12.3	H_2O (pH 7.0)	cal	[31]
202	triiodide (anion)	40.5	49.4	7.9	H_2O (pH 11.0)	cal/uv	[31]
203	triethylamine	19.9	14.2	10.5	H_2O	cal	[31]
204	triprolidine·HCl	20.0	14.3	10.6	H_2O (pH 7.0)	cal	[31]
205	*L*-tryptophan (anion)	18.3	8.0	13.0	H_2O (pH 11.3)	cal	[31]
206	*L*-tryptophan	17.3	9.0	11.4	H_2O	cal	[31]
207	*L*-trypthophan (cation)	19.1	13.6	10.1	0.1 M H_2SO_4	uv	[43]
208	*p*-toluenesulfonate (anion)	16.5	11.3	9.0	H_2O	uv	[42]
209	undecanedioate	28.3	22.7	13.3	H_2O (pH 11.3)	cal	[31]
210	undecanedioate	28.4	25.1	11.8	D_2O (pD 13)	nmr	[31]

[a] cal – calorimetry; fl – fluorimetry; kin – kinetics (competitive rate constant determination or stopped flow); lc – high-performance liquid chromatography; pot – potentiometry; uv – spectrophotometry; nmr – nuclear magnetic resonance.

References

1. Bolmatenkov, D.N.; Nizamov, I.I.; Solomonov, B.N.; Yagofarov, M.I. Chemical Databases as a Promising Tool for Obtaining the Vaporization Characteristics of Organic Compounds. *Journal of Chemical & Engineering Data* **2025**, *70*, 4351-4361, doi:10.1021/acs.jced.5c00506.
2. Nagrimanov, R.N.; Ibragimova, A.R.; Italmasov, A.R.; Kornilov, D.A.; Ziganshin, M.A.; Solomonov, B.N.; Verevkin, S.P. Energetics of substituent effects on the benzene ring: CH3O with F, Cl, Br, and I. *Journal of Thermal Analysis and Calorimetry* **2023**, *148*, 1087-1108, doi:10.1007/s10973-022-11673-1.
3. Nagrimanov, R.N.; Zaitsau, D.H.; Abdullah, R.S.; Blokhin, A.V.; Solomonov, B.N. Thermochemistry of formation and phase transitions of substituted thiophenes at 298.15 K. *The Journal of Chemical Thermodynamics* **2023**, *186*, 107123, doi:https://doi.org/10.1016/j.jct.2023.107123.
4. Verevkin, S.P.; Nagrimanov, R.N.; Turovtsev, V.V. Structure-property relationships in alkoxy substituted benzoates from experimental and computational thermochemistry. *Chemical Thermodynamics and Thermal Analysis* **2023**, *12*, 100123, doi:https://doi.org/10.1016/j.ctta.2023.100123.
5. Bolmatenkov, D.N.; Nizamov, I.I.; Aleshin, R.P.; Galukhin, A.V.; Yagofarov, M.I.; Solomonov, B.N. Vaporization thermodynamics of normal alkyl azides. *Thermochimica Acta* **2024**, *740*, 179840, doi:https://doi.org/10.1016/j.tca.2024.179840.
6. Notfullin, A.A.; Bolmatenkov, D.N.; Yagofarov, M.I.; Balakhontsev, I.S.; Ziganshin, M.A.; Solomonov, B.N. Vaporization thermodynamics of normal alkyl benzoates. *Journal of Thermal Analysis and Calorimetry* **2022**, *147*, 14631-14647.
7. Samatov, A.A.; Konyashin, K.S.; Solomonov, B.N.; Nagrimanov, R.N. Influence of the position of carbonyl group in ketones on their enthalpies of vaporization and saturated vapour pressures. *Journal of Thermal Analysis and Calorimetry* **2024**, *149*, 14913-14924, doi:10.1007/s10973-024-13816-y.
8. Růžička, K.; Štejfa, V.; Červinka, C.; Fulem, M.; Šturala, J. Thermodynamic Study of N-Methylformamide and N,N-Dimethyl-Formamide. *Molecules* **2024**, *29*, 1110.
9. Piltan, A.; Štejfa, V.; Fulem, M.; Růžička, K. Thermophysical Properties for Alkylphosphonate and Alkylphosphate Compounds. *International Journal of Thermophysics* **2024**, *45*, 59, doi:10.1007/s10765-024-03349-9.
10. Zalčík, A.; Štejfa, V.; Fulem, M.; Růžička, K. Vapor pressures and thermophysical properties of selected monoterpenoids II. *Fluid Phase Equilibria* **2023**, *567*, 113704.
11. Almeida, A.R.R.P.; Pinheiro, B.D.A.; Monte, M.J.S. Research on the volatility, phase transitions and thermodynamic stability of five organochlorine compounds. *Chemosphere* **2024**, *351*, 141224, doi:https://doi.org/10.1016/j.chemosphere.2024.141224.
12. Almeida, A.R.R.P.; Pinheiro, B.D.A.; León, G.P.; Postolnyi, B.; Araújo, J.P.; Monte, M.J.S. Exploring the Volatility, Phase Transitions, and Solubility Properties of Five Halogenated Benzaldehydes. *Molecules* **2025**, *30*, 1551.
13. Kazakov, A.; Paulechka, E.; Chirico, R.D. Reconciliation of Experimental and Computed Thermodynamic Properties for Methyl-Substituted 3-Ring Aromatics. Part 1: 9-Methylanthracene. *Journal of Chemical & Engineering Data* **2022**, *67*, 1834-1850, doi:10.1021/acs.jced.1c00907.
14. Bolmatenkov, D.N.; Nizamov, I.I.; Sokolov, A.A.; Notfullin, A.A.; Solomonov, B.N.; Yagofarov, M.I. Polymorphism and Phase-Transition Thermodynamic Properties of Phenazone (Antipyrine). *Molecules* **2025**, *30*, 2814.
15. Morecroft, D.W. Vapor Pressures of Some High Molecular Weight Hydrocarbons. *Journal of Chemical & Engineering Data* **1964**, *9*, 488-490, doi:10.1021/je60023a003.

16. Roháč, V.; Čenský, M.; Zala, D.; Růžička, V.; Růžička, K.; Sporka, K.; Aim, K. Vapor Pressure and Liquid Heat Capacity of Perhydroacenaphthylene and Perhydrophenanthrene. *Journal of Chemical & Engineering Data* **2000**, *45*, 1205-1210, doi:10.1021/je000130n.
17. https://www.nist.gov/mml/csd/chemical-informatics-research-group/products-and-services/fourier-grid-hamiltonian-fgh-1d. Available online: (accessed on 28 September 2026).
18. Moine, E.; Privat, R.; Sirjean, B.; Jaubert, J.-N.; Müller, S.; Marques, H. Erratum: "Estimation of Solvation Quantities from Experimental Thermodynamic Data: Development of the Comprehensive CompSol Databank for Pure and Mixed Solutes" [J. Phys. Chem. Ref. Data 46, 033102 (2017)]. *Journal of Physical and Chemical Reference Data* **2024**, *53*, doi:10.1063/5.0235083.
19. Sedov, I.A.; Stolov, M.A.; Solomonov, B.N. Solvophobic effects and relationships between the Gibbs energy and enthalpy for the solvation process. *Journal of Physical Organic Chemistry* **2011**, *24*, 1088-1094, doi:https://doi.org/10.1002/poc.1859.
20. Solomonov, B.N.; Yagofarov, M.I. Compensation relationship in thermodynamics of solvation and vaporization: Features and applications. II. Hydrogen-bonded systems. *Journal of Molecular Liquids* **2023**, *372*, 121205, doi:https://doi.org/10.1016/j.molliq.2023.121205.
21. Solomonov, B.N.; Khisamiev, M.B.; Yagofarov, M.I. Compensation Relationships in the Solvation Thermodynamics of Proton Acceptors in Aliphatic Alcohols. *Liquids* **2025**, *5*, 17.
22. Sedov, I.A.; Stolov, M.A.; Solomonov, B.N. Thermodynamics of solvation and solvophobic effect in formamide. *Journal of Chemical Thermodynamics* **2013**, *64*, 120-125, doi:10.1016/j.jct.2013.05.006.
23. Sedov, I.A.; Magsumov, T.I.; Stolov, M.A.; Solomonov, B.N. Standard molar Gibbs free energy and enthalpy of solvation of low polar solutes in formamide derivatives at 298K. *Thermochimica Acta* **2016**, *623*, 9-14, doi:https://doi.org/10.1016/j.tca.2015.11.010.
24. Sedov, I.A.; Stolov, M.A.; Solomonov, B.N. Enthalpies and Gibbs free energies of solvation in ethylene glycol at 298K: Influence of the solvophobic effect. *Fluid Phase Equilibria* **2013**, *354*, 95-101, doi:10.1016/j.fluid.2013.06.028.
25. Sedov, I.A.; Stolov, M.A.; Solomonov, B.N. Thermodynamics of solvation in propylene glycol and methyl cellosolve. *Journal of Chemical Thermodynamics* **2014**, *78*, 32-36, doi:10.1016/j.jct.2014.06.006.
26. Katritzky, A.R.; Tulp, I.; Fara, D.C.; Lauria, A.; Maran, U.; Acree, W.E. A general treatment of solubility. 3. Principal component analysis (PCA) of the solubilities of diverse solutes in diverse solvents. *Journal of chemical information and modeling* **2005**, *45*, 913-923.
27. Mintz, C.; Ladlie, T.; Burton, K.; Clark, M.; Acree Jr, W.E.; Abraham, M.H. Enthalpy of solvation correlations for gaseous solutes dissolved in alcohol solvents based on the Abraham model. *QSAR & Combinatorial Science* **2008**, *27*, 627-635.
28. Sprunger, L.M.; Achi, S.S.; Acree Jr, W.E.; Abraham, M.H. Development of correlations for describing solute transfer into acyclic alcohol solvents based on the Abraham model and fragment-specific equation coefficients. *Fluid phase equilibria* **2010**, *288*, 139-144.
29. Abraham, M.H.; Acree Jr, W.E.; Cometto-Muñiz, J.E. Partition of compounds from water and from air into amides. *New Journal of Chemistry* **2009**, *33*, 2034-2043, doi:10.1039/B907118K.
30. Schmidt, A.; Zad, M.; Acree, J.W.E.; Abraham, M.H. Development of Abraham model correlations for predicting enthalpies of solvation of nonionic solutes dissolved in formamide. *Physics and Chemistry of Liquids* **2016**, *54*, 313-324, doi:10.1080/00319104.2015.1084882.

31. Rekharsky, M.V.; Inoue, Y. Complexation Thermodynamics of Cyclodextrins. *Chemical Reviews* **1998**, *98*, 1875-1918, doi:10.1021/cr970015o.
32. Brewster, R.E.; Teresa, B.F.; Schuh, M.D. Inclusion Complexes of 6-Bromo-2-Naphthol (Guest) and α-Cyclodextrin (Host): Thermodynamics of the Binary Complex and First-Reported Dynamics of a Triplet-State Guest/Host2 Complex. *The Journal of Physical Chemistry A* **2003**, *107*, 10521-10526, doi:10.1021/jp030253p.
33. Buschmann, H.-J.; Schollmeyer, E. Influence of the Solvation Upon the Reaction of α-Cyclodextrin with Carboxylic Acids, Their Methyl Esters, and Their Sodium Salts in Aqueous Solution Studied by Calorimetric Measurements. *Journal of Solution Chemistry* **2005**, *34*, 731-737, doi:10.1007/s10953-005-4498-x.
34. Hamai, S.; Takahashi, A.; Hori, K. Thermodynamic and 13C NMR Studies of the Inclusion Complexes of α- and β-Cyclodextrins with Cyano- and Nitrophenols in Aqueous Solution. *Journal of inclusion phenomena and macrocyclic chemistry* **2000**, *37*, 197-207, doi:10.1023/A:1008199921925.
35. Di Marino, A.; Mendicuti, F. Thermodynamics of Complexation of Dimethyl Esters of Tere-, Iso-, and Phthalic Acids with α- and β-Cyclodextrins. *Applied Spectroscopy* **2004**, *58*, 823-830, doi:10.1366/0003702041389283.
36. Alvariza, C.; Usero, R.; Mendicuti, F. Binding of dimethyl 2,3-naphthalenedicarboxylate with α-, β- and γ-cyclodextrins in aqueous solution. *Spectrochimica Acta Part A: Molecular and Biomolecular Spectroscopy* **2007**, *67*, 420-429, doi:https://doi.org/10.1016/j.saa.2006.07.039.
37. Pastor, I.; Marino, A.D.; Mendicuti, F. Complexes of dihexyl 2,6-naphthalenedicarboxylate with α- and β-cyclodextrins: Fluorescence and molecular modelling. *Journal of Photochemistry and Photobiology A: Chemistry* **2005**, *173*, 238-247, doi:https://doi.org/10.1016/j.jphotochem.2005.04.003.
38. Qu, X.K.; Sun, D.Z.; Liu, F.; Wei, X.L.; Wang, Z.N.; Li, G.Z. Study on inclusion complexes of cyclodextrin with sodium dodecyl polyoxyethylenated sulfonate using microcalorimetry and 1H NMR. *Journal of dispersion science and technology* **2007**, *28*, 779-784.
39. Castronuovo, G.; Niccoli, M. Solvent effects on the complexation of 1-alkanols by parent and modified cyclodextrins. Calorimetric studies at 298 K. *Journal of Thermal Analysis and Calorimetry* **2011**, *103*, 641-646, doi:10.1007/s10973-010-0965-8.
40. Buschmann, H.-J.; Mutihac, L.; Schollmeyer, E. Thermodynamic Data for the Complex Formation of Alkylamines and Their Hydrochlorides with α-Cyclodextrin in Aqueous Solution. *Journal of inclusion phenomena and macrocyclic chemistry* **2005**, *51*, 53-57, doi:10.1007/s10847-004-6093-y.
41. Buschmann, H.J.; Dong, H.; Schollmeyer, E. Complexation of Aliphatic Alcohols by α- and β-Cyclodextrins and their Partial Methylated Derivatives in Aqueous Solution. *Journal of Thermal Analysis and Calorimetry* **2000**, *61*, 23-28, doi:10.1023/A:1010140019557.
42. Ondo, D.; Tkadlecová, M.; Dohnal, V.; Rak, J.; Kvíčala, J.; Lehmann, J.K.; Heintz, A.; Ignatiev, N. Interaction of Ionic Liquids Ions with Natural Cyclodextrins. *The Journal of Physical Chemistry B* **2011**, *115*, 10285-10297, doi:10.1021/jp205039g.
43. Yuexian, F.; Yu, Y.; Shaomin, S.; Chuan, D. Molecular recognition of α-cyclodextrin (CD) to choral amino acids based on methyl orange as a molecular probe. *Spectrochimica Acta Part A: Molecular and Biomolecular Spectroscopy* **2005**, *61*, 953-959, doi:https://doi.org/10.1016/j.saa.2004.06.009.
44. Marino, A.D.; Rubio, L.; Mendicuti, F. Fluorescence and Molecular Mechanics of 1-Methyl Naphthalenecarboxylate/Cyclodextrin Complexes in Aqueous Medium. *Journal of*

Inclusion Phenomena and Macrocyclic Chemistry **2007**, *58*, 103-114, doi:10.1007/s10847-006-9129-7.

45. Castronuovo, G.; Elia, V.; Niccoli, M.; Velleca, F. The Effects of Cosolvents on the Complexation of α-Cyclodextrin with Alkylated Substances. Calorimetric Studies at 25 °C. *Journal of inclusion phenomena and macrocyclic chemistry* **2002**, *44*, 229-234, doi:10.1023/A:1023019118389.
